# Parylenes in bioanalytical and cell culture applications

**DOI:** 10.1007/s00216-026-06682-0

**Published:** 2026-07-23

**Authors:** Florian Bender, Kerstin Länge

**Affiliations:** 1 Department of Electrical and Computer Engineering, Marquette University, Milwaukee, WI 53233 USA; 2 Institute of Microstructure Technology, Karlsruhe Institute of Technology, Eggenstein-Leopoldshafen, 76344 Germany

**Keywords:** Parylene, diX, Surface functionalization, Cell culture, Biosensors, Bioassays

## Abstract

Parylenes are a family of polymers derived from poly(*p*-xylylene). The most commonly used member of this family is parylene C, which contains one chlorine substituent per monomer unit. It is used as a passivation layer in bioanalytical devices, among other applications. However, parylene C is chemically inert. This limits its use in bioanalytical applications and other fields that require chemical functionalization of the surface. Therefore, various methods have been developed to functionalize parylene C surfaces. Other approaches have aimed to develop parylenes with other moieties that are easier to functionalize. Depending on their functional group, parylenes have different characteristics, fields of application, and adjustment possibilities. In this work, four groups of parylenes are discussed: (1) parylene C and parylene N (poly(*p*-xylylene)) as the most common representatives of this polymer family, (2) heat-resistant parylenes, which include fluorinated parylenes, (3) parylenes with functional groups for protein coupling, such as amino groups and formyl groups, and (4) parylenes with moieties for click chemistry, allowing for a wide variety of functionalization and application. Applications include cell culture, biosensors, immunoassays, and enzyme assays, among others. A comparison of the performance of the respective parylenes is made, if possible, with regard to a specific analytical task. Though many tasks have been accomplished with available parylenes, limitations regarding functional groups, including the ability to undergo functionalization, still exist. This paves the way for new parylenes or new methods of surface modification that have not yet been widely adopted and are ready to be investigated.

**Graphical abstract:**

**Figure:**
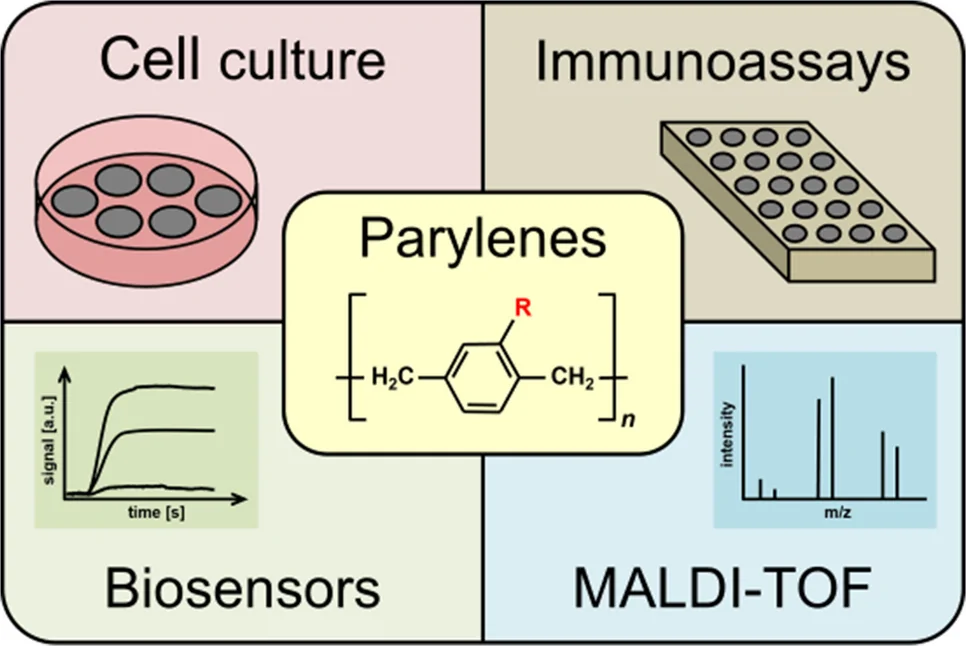

## Introduction

**Fig. 1:**
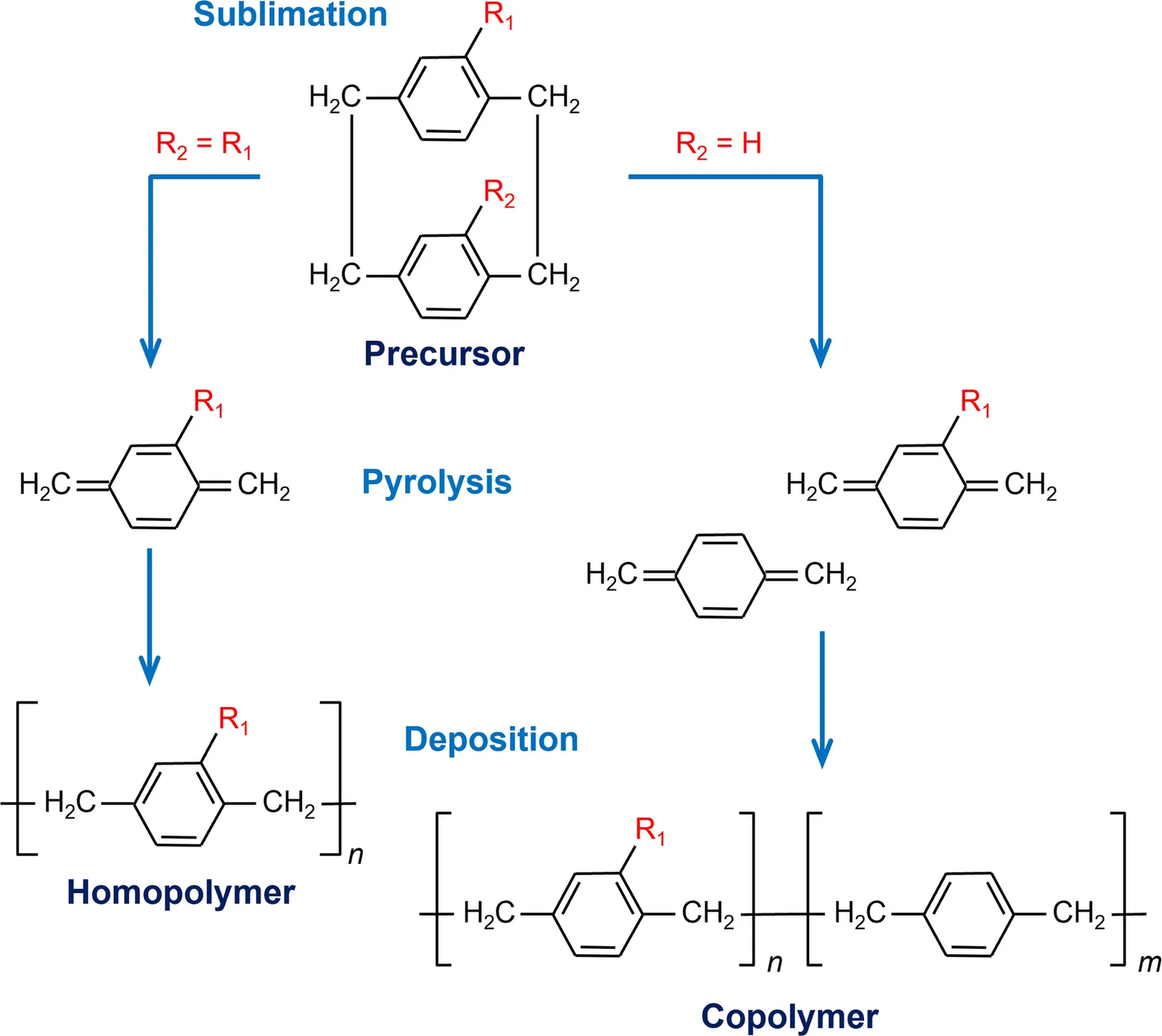
Gorham process for the deposition of parylene (non-stoichiometric)

Poly(*p*-xylylenes) are polymers made from [2.2] paracyclophanes via chemical vapor deposition (CVD). While the term “poly(*p*-xylylene)” is sometimes abbreviated as “PPX,” the term “parylene” is much more common for this class of polymers [1]. The CVD process developed by Gorham for the deposition of parylene layers is depicted in Fig. 1 [2, 3]. First, the precursor is sublimated at temperatures between 120 and 160 °C and a pressure of 1 mbar. Then, it is pyrolyzed at temperatures between 650 and 700 °C and a pressure of 0.5 mbar, resulting in reactive monomers. In the deposition zone, the monomer polymerizes near room temperature at a pressure of 0.1 mbar [4]. The exact process parameters depend on which precursor is used, while the layer thickness is mainly determined by the amount of precursor applied in the process [2, 3, 5–7].

The parylene deposition process typically produces high-molecular-weight linear polymer chains that form semi-crystalline parylene films [2, 8]. In the CVD process, the conventional method of cracking the gaseous precursor is thermal pyrolysis. However, the use of plasma in this step has also been investigated because it allows for a faster CVD process. Parylene layers obtained with plasma deposition exhibited lower contact angles than the parylene layers obtained by thermal deposition since the functional groups on the surface were partly complemented by oxygen- and nitrogen-containing species. Furthermore, plasma-deposited parylenes were more amorphous than the thermally deposited counterparts [4, 9, 10]. The crystallinity of parylene films is influenced by both pyrolysis and deposition parameters [7, 9]. While annealing the films after CVD can improve their crystallinity, it can also negatively affect their adhesion to underlying materials [11, 12]. The roughness of parylene films is also influenced by the deposition parameters of the CVD process [1, 11]. If required, the roughness can be increased by treatment with physical plasma after the CVD [13].

Table 1 lists the names of the most common commercially available parylenes. These are homopolymers, i.e., the underlying precursors are dimers consisting of two identical monomers. This results in the CVD process shown on the left in Fig. 1. The molecular structures of the parylenes are presented in the subsequent chapters.

**Table 1:** Overview of commonly available parylenes [14–16]

Name	Alternative name	Functional groups	Molecular formula of dimer	CAS RN of dimer
Parylene N	diX, PPX	None	C_16_H_16_	1633-22-3
Parylene C	diX-C, PPX-C	One Cl substituent per benzene ring	C_16_H_14_Cl_2_	28804-46-8^a^ 10366-05-9
Parylene D	diX-D, PPX-D	Two Cl substituents at the 2,5-position of each benzene ring	C_16_H_12_Cl_4_	30501-29-2^a^ 25826-07-7
Parylene AF4	Parylene F-AF4, Parylene HT®^b^, Parylene SF, diX-SF	Fluorinated methylene groups (CF_2_) between the benzene rings	C_16_H_8_F_8_	3345-29-7
Parylene VT4	Parylene F, Parylene F-VT4, Parylene CF	Fluorinated benzene rings	C_16_H_8_F_8_	1785-64-4

^a^Incompletely defined substance, i.e., the position of the chlorine substituents is unknown [17, 18]

^b^Trademarked by Specialty Coating Systems (SCS) [19]

The name “parylene” was originally introduced in the USA by Union Carbide Corporation (UCC) and later transferred to Specialty Coating Systems (SCS), along with all related information and patents [2, 20]. In Japan, Daisan Kasei Co., a partner company of Kisco, registered the brand name “diX” for parylenes [21, 22]. “PPX” stands for “poly(*p*-xylylene)” [1]. The name “parylene F” is usually associated with the parylene containing fluorinated benzene rings (parylene VT4 in Table 1). However, in some publications, “parylene F” refers to the parylene with fluorinated methylene groups (CF_2_) between the benzene rings (parylene AF4 in Table 1) [23]. To avoid any ambiguity, the names “parylene AF4” and “parylene VT4” (as depicted in Table 1) are used in the following. Two Chemical Abstracts Service Registry Numbers (CAS RNs) are listed for both parylene C and parylene D. The first CAS RNs refer to substances that are not fully defined, meaning the position of the chlorine substituents is unknown [17, 18]. The second CAS RNs refer to the actual dimer. However, both CAS RNs are used by suppliers of these parylene dimers.

CVD of common parylenes (Table 1) results in thin, conformal and stress-free layers. The layers are optically transparent, electrically insulating, impermeable for gas and moisture, and biocompatible. Due to this unique combination of properties within one polymer family, parylenes are ideal for use as protective or functional coatings in sensors and electronic devices, particularly in bioanalytical applications. However, the parylenes listed in Table 1 are considered chemically inert. This limits their use in applications that require chemical functionalization of the surface [8, 24]. Other parylenes exhibit reactive moieties, such as alkynyl groups (mostly ethynyl), amino groups, anhydride groups, benzoyl groups, carbonyl groups, ester groups, formyl (aldehyde) groups, and hydroxyl groups. These moieties enable the covalent immobilization of biomolecules, such as proteins or saccharides, on the parylene surface. Reactions include the activation of carboxyl groups via 1-Ethyl-3-(3-dimethylaminopropyl)carbodiimide and N-hydroxysuccinimide (EDC/NHS) to form peptide bonds with amino groups, the formation of Schiff bases between amino and aldehyde groups, and click chemistry between alkynyl and azide groups [1, 3, 25–28]. Furthermore, poly(4-benzoyl-*p*-xylylene-*co*-*p*-xylylene), a photoreactive parylene providing benzoyl groups, was reported to have the properties of a photoresist. This allows for the photopatterning of biointerfaces without the use of solvents or irradiation, which could potentially damage sensitive biomolecules. It also enables the presentation of biomolecules on substrates with complex geometries. This approach could be useful for fabricating tissue engineering scaffolds, three-dimensional (3D) cell cultures, and next-generation bioMEMS devices [27].

As only a limited number of parylenes with reactive moieties have been commercially available, they are typically synthesized from scratch using the correspondingly functionalized [2.2]paracyclophanes, which also may have to be prepared first. Furthermore, as previously mentioned, the parameters of the CVD process must be adjusted according to the respective precursor [2, 3, 5, 7], which can be time-consuming and requires additional material.

An alternative would be to introduce functional groups to commonly available parylenes (Table 1), for which the deposition parameters are well-known. One method would be oxidation. Since these parylenes are mostly chemically inert, strong oxidizing agents are required, such as piranha solution [29], a mixture of concentrated hydrogen peroxide (H₂O₂) and concentrated sulfuric acid (H_2_SO_4_). Oxidation with piranha solution requires careful handling and thorough cleaning afterward due to the highly aggressive nature of the chemicals involved. Physical oxidation methods are mostly residue-free, making them easier to handle. These methods include thermal oxidation [30, 31], photooxidation [32, 33], and oxygen (or atmospheric) plasma treatment [34–36]. However, thermal oxidation can cause cracking of the material [31] and is therefore rarely used for parylene surface treatment. Better results are obtained with photooxidation by UV exposure or plasma treatment. The oxidized parylene surfaces were used directly, taking advantage of their increased hydrophilicity compared to the untreated parylene surfaces [37–40]. Alternatively, they were converted by reacting with other chemicals, such as silanes, to enable further covalent coupling [41].

Other methods for chemically functionalizing parylene layers include electrophilic aromatic substitution reactions, such as the Friedel–Crafts acylation [42], and the Buchwald–Hartwig amination. The latter was applied for the palladium-catalyzed substitution of the chlorine substituent of parylene C by an amino group [43]. Parylene C that underwent Friedel–Crafts acylation was further functionalized to improve protein immobilization and cell and tissue adhesion [44, 45]. A fast and gentle introduction of dextran layers, which were used as part of biosensing layers, to parylenes was achieved through photochemical reactions based on aryldiazirine-labeled compounds [46, 47]. Although the reactions proceeded rapidly and gently, the compounds were not directly available and had to be synthesized beforehand. A recent study introduced the photo-Arbuzov reaction to halogenated parylenes, such as parylene C, in order to obtain phosphonated surface coatings. Subsequent phosphorus-based surface chemistry may lead to a new set of coatings that can be used for biosensing applications and tissue engineering [48].

The following summarizes commonly used parylenes and their applications in bioanalytics and cell culture, including surface modifications, if required. While the focus is on the chemical microenvironment of the parylenes and their potential functionalization, the specific properties of parylenes that allow for particular applications, such as insulating layers or acoustic waveguiding layers, are also addressed.

The parylenes covered here are as follows:

- Parylene C and parylene N, the most common parylenes [8]
- Parylene D, parylene AF4, and parylene VT4, which are more heat-resistant
- Parylene A, parylene AM, and parylene H, which provide functional groups that are ideal for protein coupling
- Parylene X and other parylenes with functional groups allowing click chemistry

Bioanalytical applications, apart from cell culture, mainly include biosensor applications, immunoassays, and enzyme assays. The characteristics of each type of parylene are explained, as well as how these characteristics can be used in specific applications.

## Applications of parylenes

### The most common parylenes: parylene C and parylene N

#### The most common parylenes: overview

**Fig. 2:**
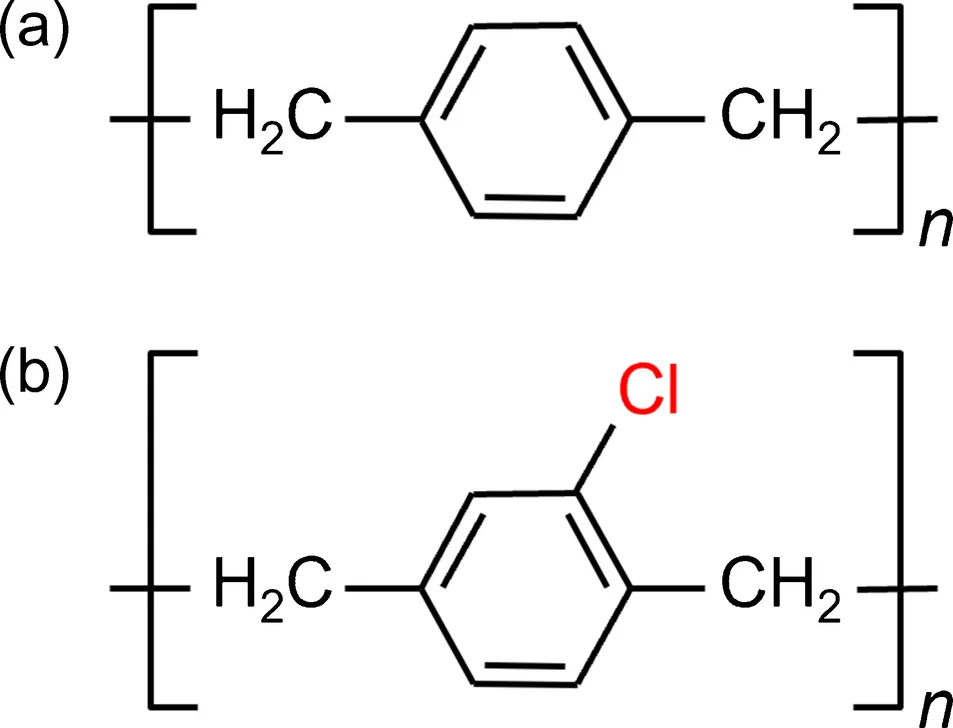
Molecular structure of **a** parylene N and **b** parylene C

Parylene C is by far the most common type of parylene, followed by parylene N, the unsubstituted base form [8]. Figure 2 shows the molecular structures of these parylenes, which exist as homopolymers.

Both parylene C and parylene N have been used with and without further functionalization. The applications shown below use one or both of these parylenes. Applications involving other parylenes, compared to parylene C or parylene N, are listed in the sections dedicated to the respective parylenes.

#### The most common parylenes in cell culture

Parylene C and parylene N form dense, smooth, and highly reproducible coatings that represent effective molecular barriers even on 3D scaffolds. Therefore, these parylenes can provide protection of sensitive cell or tissue cultures from cytotoxic materials commonly used in 3D-printed resins in organ-on-chip and cell culture devices [24, 49]. Both parylene C and parylene N have been rated a United States Pharmacopeia (USP) Class VI polymer because of their favorable properties, including high biocompatibility, biostability, low cytotoxicity, and resistance against hydrolytic degradation. Parylene C, in particular, is frequently used in the medical industry as a coating material [8, 24]. Consequently, cell adsorption on both parylene C and parylene N surfaces has been the subject of various studies.

**Fig. 3:**
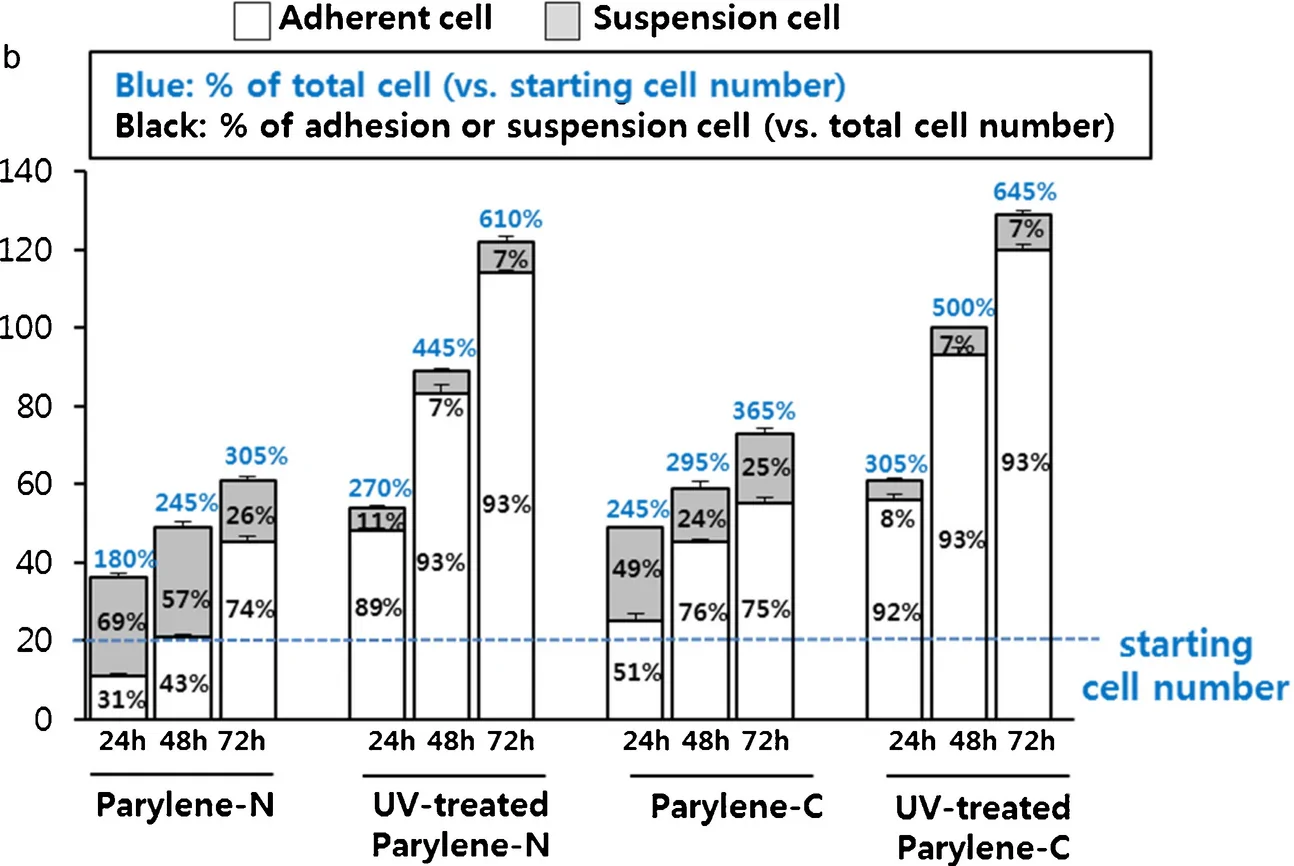
PC-12 cells cultivated on parylene N and parylene C films, both with and without UV treatment, with an initial seeding of 20,000 cells per plate. The percentage data above the columns represents the increase in cell number during cultivation. The percentage data in the white and grey sections of the columns represent the ratio of adherent to suspended cells, respectively. Reprinted from *Enzyme Microb. Technol.* 2017, 97:1–10. Copyright 2017 Elsevier

The influence of surface oxidation on surface wettability and, consequently, cell adhesion [50] has frequently been investigated using parylene C and parylene N. Oxidation makes parylene N and parylene C surfaces more hydrophilic, creating a different microenvironment that may affect cell adhesion depending on the cell type. Physical oxidation methods, such as UV and plasma oxidation, were preferred due to their simpler handling and residue-free nature compared to chemical methods. Osteoblast-like MG-63 cells and neuron-like PC-12 cells both adhered better to the surfaces of parylene N and parylene C after they were oxidized through UV exposure (Fig. 3) [37, 38].

For parylene C and both fibroblast and hepatocyte cell lines, it was found that the untreated polymer minimizes cell adhesion while plasma treatment of the parylene C coating leads to increased cell adhesion. The increased cell adhesion was comparable to that obtained with commercially available tissue culture-treated polystyrene [51]. Similar results were obtained with PC-12 cells [39], osteosarcoma cells [13], and human umbilical vein endothelial cells (HUVECs) [52] on parylene C with and without plasma treatment.

Parylene N prepared through plasma polymerization exhibited higher hydrophilicity than parylene N resulting from thermal deposition. A higher fibroblast cell count was obtained with plasma-polymerized parylene N, consistent with results obtained with plasma-treated, conventionally deposited parylene N. Since no post-modification is required, plasma-polymerized parylenes show immediate promise for cell adhesion [53].

#### The most common parylenes in acoustic-wave biosensors

Acoustic-wave biosensors mainly use quartz crystal microbalance (QCM) and surface acoustic wave (SAW) devices as detectors [54]. Parylenes have many potential applications in acoustic-wave sensors. Due to their dense structure and mechanical properties, parylenes can be used as protective coatings for the electrical shielding of electrode structures. Furthermore, parylenes are particularly important for SAW biosensors because they can increase the sensitivity of the SAW sensor response to analyte binding when used as waveguide materials. Additionally, the conformality of parylenes makes them useful as an initial layer for chemically homogenizing 3D surfaces. This is useful for preparing surfaces for the covalent immobilization of bioreceptor molecules or other compounds. Alternatively, parylenes can serve as a surface for non-specific protein or cell adsorption. Examples of the main uses of parylenes in acoustic-wave biosensors are given below.

QCM devices consist of a thin, circular quartz wafer sandwiched between two metal-coated electrodes [54]. Forming a cavity with a parylene diaphragm attached to one of the QCM electrodes via parylene microposts reduces the damping effect of liquids on the QCM surface. This enables a QCM sensor design that performs better in liquids [55]. QCM sensors coated with parylene C were used to measure the activated partial thromboplastin time (aPTT) and monitor blood coagulation [56, 57]. QCM sensors coated with parylene N were investigated for monitoring *Escherichia coli* (*E. coli*) populations [58]. The studies made use of the hydrophobic nature of both parylenes, meaning that they were not further functionalized.

SAW devices consist of a piezoelectric substrate with interdigitated metal electrodes on its surface [54]. Parylene C was observed to provide efficient electrical shielding and chemical protection of aluminum electrodes or bond wires against corrosive attack in SAW biosensor measurements [46, 59]. A thickness of 35 nm was determined to be the minimum value needed to achieve continuity and effective protection by parylene C coatings [6]. Additionally, parylene C coatings enabled capacitive coupling between SAW devices and circuit boards, eliminating the need for bond wires [60]. A combination of a parylene C coating and a gold layer was used to demonstrate effective elimination of acoustoelectric interaction between conducting solutions and electrode structures [61].

It has long been known that polymers can be used to confine the energy of shear-horizontal (SH)-SAW sensors to a surface. This creates a sensor with high sensitivity to surface perturbation that can be used for direct liquid-phase measurement [62, 63]. The waveguiding effect was confirmed for parylene C-coated SH-SAW sensors. The resulting wave type is also referred to as “Love Mode” [64, 65]. Under liquid-phase conditions, parylene C waveguides were found to increase mass sensitivity by up to a factor of 8.6 [64]. Furthermore, parylene C waveguides were found to increase sensitivity in Lamb wave biosensors [66]. This demonstrates that parylene coatings can act as waveguides, in addition to their effective electrode protection and protein immobilization abilities. Parylene C waveguides were also compared to SiO₂ waveguides. While a slightly better detection limit was found for thick (4 µm) layers of SiO₂, it was noted that SiO₂ is difficult to deposit at such thicknesses. Additionally, parylene C offers better corrosion protection for the electrodes [59]. SH-SAW sensors with a parylene C waveguide were used to detect proteins released during platelet activation [67] and to monitor the adsorption of tendon stem cells [68]. Furthermore, an antibody-coated parylene C/SiO_2_ phononic waveguide was introduced for the reliable, high-sensitivity detection of vascular endothelial growth factor (VEGF) secreted by platelets [69].

If parylene was functionalized for the aforementioned applications, adsorption was mostly used. Non-specific protein adsorption is a simple way to create a highly selective biosensor surface. The adsorbed protein then serves as the selective recognition element in a biosensor measurement. While this method does not always permit maximum sensitivity and repeatability, it is sometimes preferred due to the rapid and simple preparation of the sensor surface [46]. Additionally, non-specific sensor surfaces are used to study protein adsorption processes [70]. As demonstrated with SAW biosensors, parylene C is ideal for the non-specific adsorption of various proteins, including plasma proteins such as human serum albumin (HSA) and fibrinogen [70], bovine serum albumin (BSA) [60, 64], collagen [67], and antibodies [69].

Parylene C-coated SAW devices were covalently functionalized using custom-made aryldiazirine-labeled compounds. A dextran derivative was immobilized as an intermediate hydrogel layer to prevent non-specific protein binding in the subsequent assays. After coupling the antibody to the dextran, only the corresponding antigens bound to the SAW biosensor. Non-specific protein adsorption was largely suppressed [46, 47]. A more flexible and accessible method involves oxidizing parylene C with plasma, silanizing it, and binding a functionalized hydrogel, such as aminodextran, to the silane. This process creates suitable functional groups for immobilizing protein-based or other biomolecules. Subsequent detection of antibodies confirmed that non-specific protein adsorption was largely reduced by this functionalization method, while the corresponding binding partners resulted in a significant signal response [41]. This immobilization principle was applied to parylene C-coated SAW biosensors for the detection of breast cancer markers [71, 72], hepatocyte growth factor (HGF) in cell culture medium [73], C-reactive protein (CRP) in serum [74], and penicillin G in milk [75]. The aforementioned immobilization protocols, which are based on adsorption and covalent binding, were adapted for SAW biosensors. However, these protocols can also be transferred to other biosensors and devices that are coated with parylene.

#### The most common parylenes in bioelectronic devices

Parylene is an insulating material that becomes conductive through pyrolysis [24]. The insulating properties of parylene, in combination with the ability to form a conformal coating, result in so-called conformal insulation. This, together with its biocompatibility, makes parylene C and parylene N particularly suitable for use as coatings in bioelectronic devices, such as neural probes or implantable microelectrode arrays for the multiplexed detection of redox-active species [8, 76]. The latter was demonstrated using an array of carbon fiber multielectrodes coated with parylene, leaving the carbon fiber tips free for measurement. This array was used to detect neurotransmitters in mouse brain slices by fast-scan cyclic voltammetry [77]. Though lifetimes of several years of parylene coatings at human body temperature (37 °C) were obtained, particularly the reduced adhesion to inorganic materials remains a challenge. Studies aimed at overcoming this problem include introducing adhesion layers and multilayer approaches, with parylene serving as the top layer [12, 78].

Parylene pyrolysis can be performed at high temperatures in a furnace under a nitrogen atmosphere. Alternatively, it can be carried out using a carbon dioxide laser in either a nitrogen atmosphere or in air. The use of pyrolyzed parylene as a carbon-based material for electrochemical sensing applications was recently reviewed elsewhere [24]. A recent application describes carbon fiber microelectrodes made from pyrolyzed parylene N for the detection of amino acids and hormones in brain slices by fast-scan cyclic voltammetry [79].

#### The most common parylenes in other bioanalytical devices

An alternative biosensor design based on detection of mass and/or elastic properties is the microcantilever. Parylene C can serve as microcantilever materials that, due to their soft elastic properties, allow high sensitivity to surface stress without the necessity to use excessively thin microcantilevers [80]. Alternatively, other polymers can also be used as microcantilever materials and subsequently coated with protective layers of parylene C to increase the stability of the microcantilever in a liquid environment [81]. Microcantilevers can be replaced by thin membranes made entirely or partly of parylene, which can also serve as a sensor element sensitive to surface stress. The deflection of the membrane resulting from analyte binding can then be detected using capacitive [82] or optical [83] techniques. In the latter case, a parylene C nanosheet acting as an optical interferometric surface-stress sensor was coated with polymethyl methacrylate (PMMA). The PMMA was then oxidized using a UV/ozone cleaner, which resulted in the formation of carboxyl groups on the surface. The groups were then activated with EDC/NHS to immobilize anti-HSA antibodies. HSA detection on the functionalized nanosheet resulted in higher signal responses than obtained with other proteins [83].

Surface plasmon resonance (SPR) is a common detection principle used in optical biosensors. Gold SPR chips were coated with parylene N and treated with plasma. Antibodies were more efficiently immobilized on plasma-treated parylene N than on untreated parylene N or bare gold, demonstrating the potential of plasma-treated parylene N for protein adsorption. After the antibodies were adsorbed, the SPR biosensors were used to detect disease markers, such as the hepatitis B surface antigen (HBsAg) [84] or the inflammation marker CRP [85]. A nanoplasmonic biosensor chip that uses vertical cavity surface emitting laser (VCSEL) technology was developed for the detection of amyloid-β. An ultrathin parylene C layer, introduced as an antibody coupling layer, additionally enhanced the chip’s sensitivity [86].

#### The most common parylenes in MALDI-TOF MS

Parylene N was used to enhance the performance of matrix-assisted laser desorption/ionization time-of-flight (MALDI-TOF) mass spectrometry (MS) in the detection of compounds with a molecular weight below 1000 Da. For such analytes, the limiting factor of MALDI is that the laser ionizes parts of the matrix. The resulting peaks interfere with the analyte peaks of low-molecular-weight compounds, which makes identifying them difficult. As a preliminary step, the MALDI plates were coated with parylene N without the use of a matrix. Parylene N is more hydrophobic than the gold of standard MALDI plates, which led to improved droplet formation. This resulted in a smaller surface-to-volume ratio and a higher signal-to-noise ratio. Analytes detected in subsequent laser desorption/ionization (LDI) MS measurements included polyethylene glycol (PEG) with a molecular weight of 600 Da [87].

**Fig. 4:**
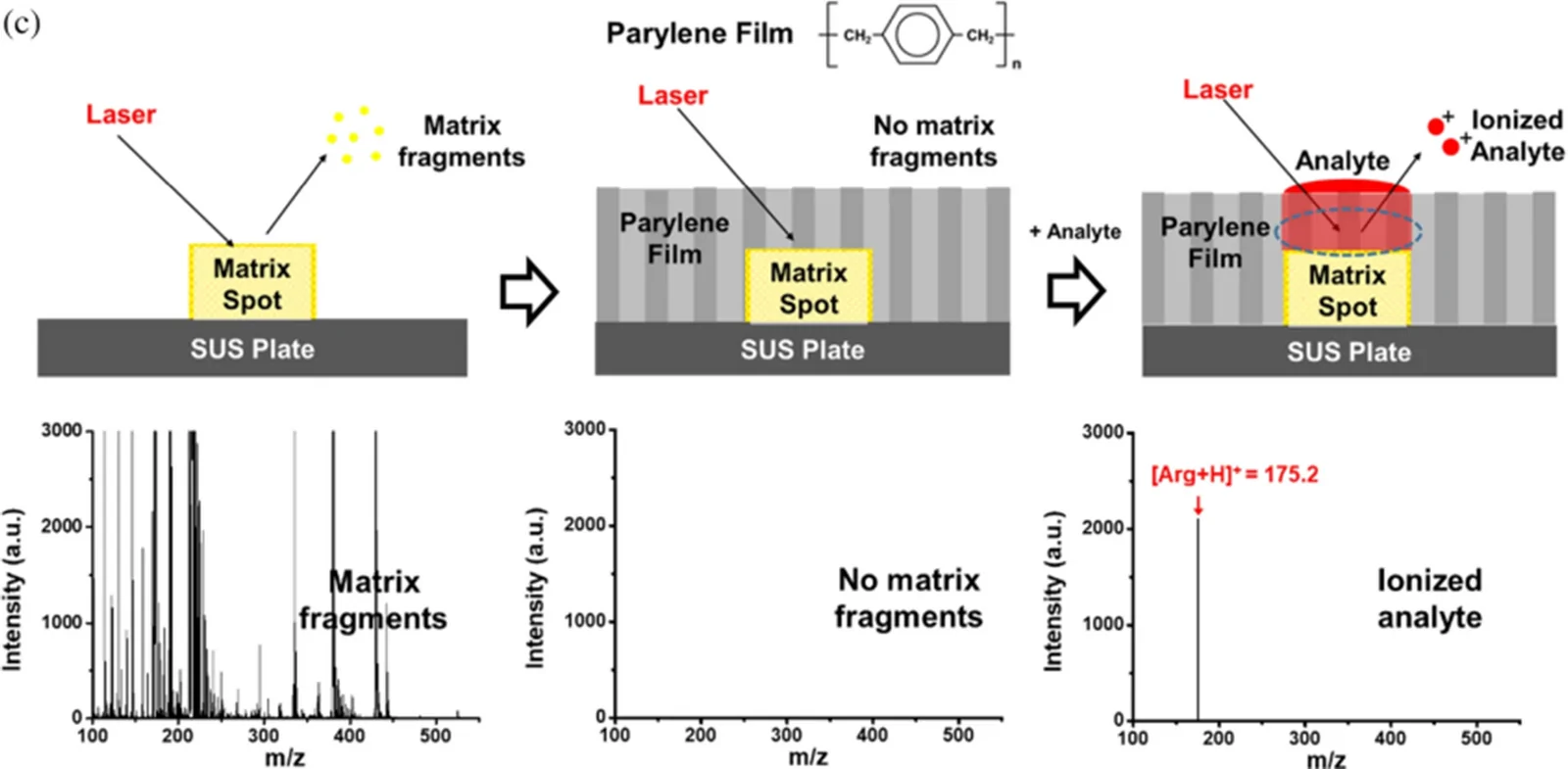
MALDI plates and mass spectra during the process. From left to right: MALDI plate coated with matrix, after coating with parylene N, and after sampling with arginine (Arg). Reprinted from *J. Anal. Sci. Technol.* 2022, 13(1):3. Published open access by Springer Nature under a CC BY license

Another method used parylene N to coat the matrix applied to the MALDI plates. The parylene N layer thickness was thin enough that the target plate’s electrical conductivity was not completely shielded, providing the required conductivity for the process. Furthermore, the parylene N layer was uniform in thickness and pinhole-free, yet slightly porous to allow the sample to interact with the matrix for MALDI-TOF detection. Mass peaks from the matrix were successfully prevented, allowing low-molecular-weight compounds to be detected with an improved signal-to-noise ratio, as shown in Fig. 4 [88].

The first analytes included amino acids and drugs with molecular weights below 500 Da [89]. This principle was used for various applications involving simultaneous detection of analytes below 1000 Da, including the detection of serum markers. Applications included enzyme assays to determine susceptibilities of bacteria strains to β-lactam antibiotics [90, 91], the detection of amino acids in serum to diagnose neonatal metabolic disorders [92], the detection of diagnostic markers for sepsis, cancer, and metabolic disorders in patient sera or spiked serum samples [93–95], and the detection of vitamin D and metabolites in serum, tablets, and energy drinks [88].

### Heat-resistant parylenes: parylene D, parylene AF4, parylene VT4

#### Heat-resistant parylenes: overview

When it comes to bioanalytical applications or cell culture, heat resistance is generally not at the top of the list of requirements. However, the manufacturing process of some devices may require thermal stability of the parylene layers, e.g., to protect electronic components [96], which is why these parylenes are included in this study. Additionally, the microenvironment provided by the functional groups has been investigated for cell culturing applications.

**Fig. 5:**
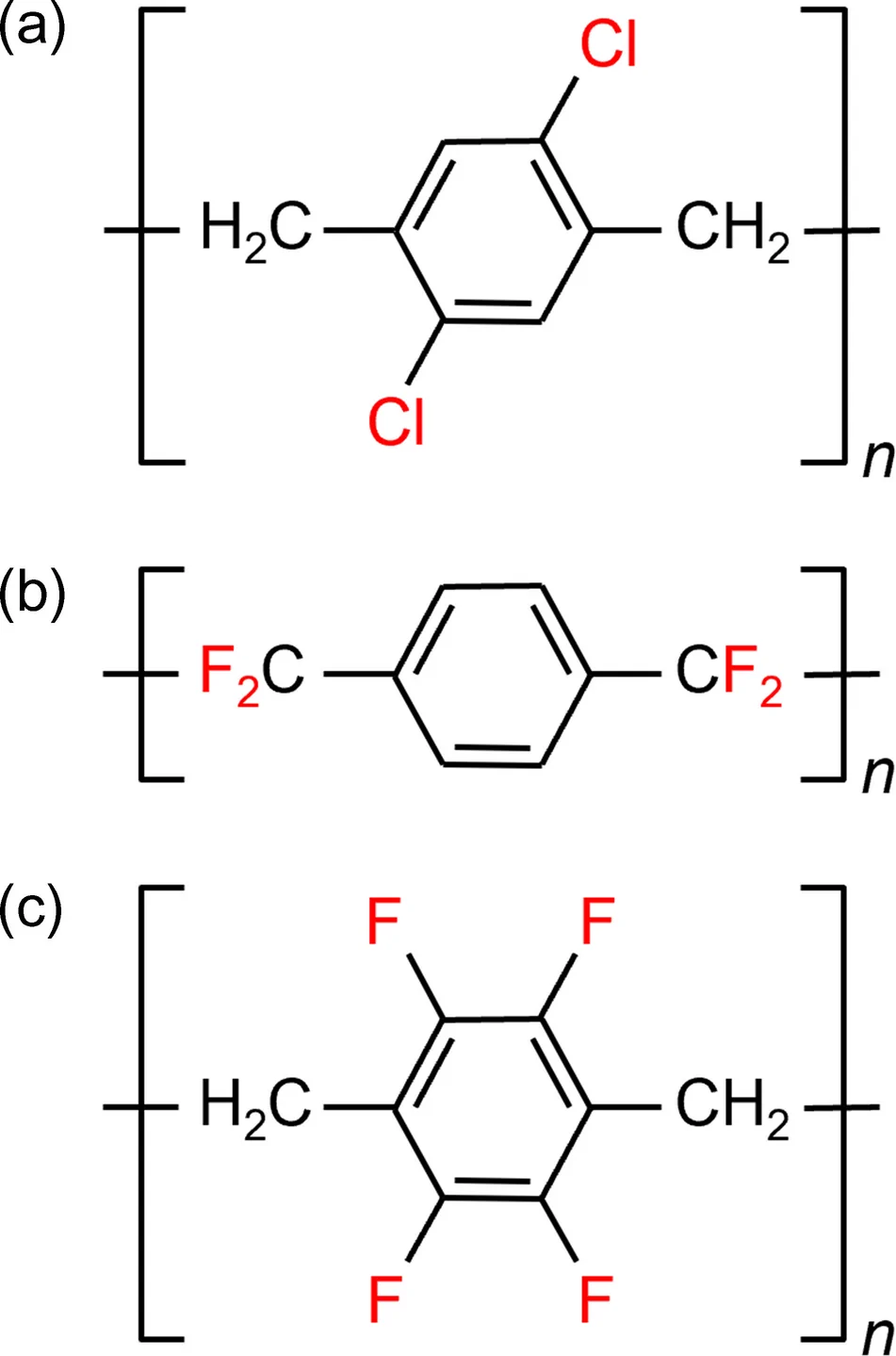
Molecular structures of **a** parylene D, **b** parylene AF4, and **c** parylene VT4

Figure 5 shows the molecular structures of parylenes that are more heat-resistant than parylene C and parylene N. They exist as homopolymers. Table 2 summarizes the parameters characterizing the thermal stability of parylenes.

**Table 2:** Thermal stability of common parylenes [97]

	N	C	D	VT4	AF4
Continuous temperature resistance (°C)	90	125	160	190	350
Temporary peak temperature (°C)	120	200	300	300	450

Parylene AF4 has the highest temperature resistance among commonly available parylenes (Table 2), and because of the fluorinated methylene groups between the benzene rings (Fig. 5b), it also has the highest stability to UV exposure. While parylene C and D exhibit similar autofluorescence behavior under UV illumination, parylene AF4 shows much lower autofluorescence, making it ideal for applications requiring sensitive fluorescence measurements [98]. However, since parylene AF4 is also the most expensive parylene variant, other parylenes are preferred, if possible [99]. Furthermore, the fluorination of the methylene groups makes the parylene AF4 dimer (and, hence, the polymer) a PFAS (per- and polyfluoroalkyl substances) candidate, in contrast to parylene VT4 carrying the fluoro groups at the benzene ring [100–102]. This requires handling parylene AF4 with particular care and consideration as it is a potential “forever chemical” [103].

#### Heat-resistant parylenes in cell culture and antifouling applications

Most applications in this section include parylene AF4. Due to its exceptional heat resistance and UV stability, it can withstand autoclaving and UV irradiation, which are common sterilization methods for cell culturing devices. Furthermore, considering the coating of the inner side of a tubing or a channel, parylene AF4 shows the highest penetrating depth and lowest water adsorption compared with parylenes N, C, and D [97, 104]. This was used to coat polydimethylsiloxane (PDMS) channels with parylene AF4 for storing aqueous droplets of several nL in oil. The droplets encapsulated bacteria, *E. coli* and *Enterococcus faecalis* (*E. faecalis*) in culture medium. Both bacteria could be grown in these droplets for several hours at 37 °C. The device was reusable after cleaning with 2-propanol, allowing at least ten uses [105].

**Fig. 6:**
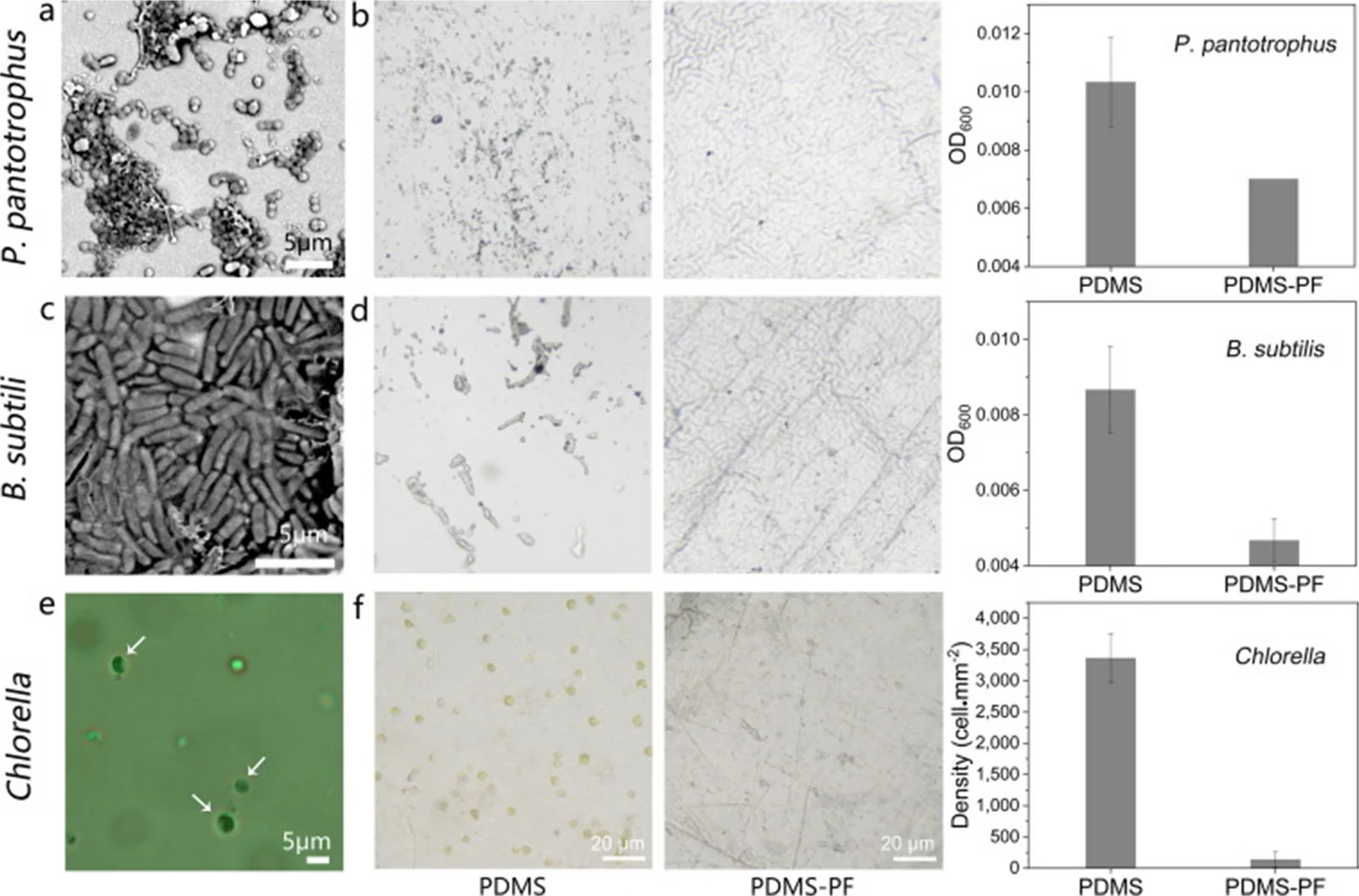
Images of bacteria and algae on silicon and glass slides, respectively, as well as adhesion assays (100-fold magnification) on PDMS and PDMS coated with parylene AF (PDMS-PF). *P. pantotrophus*: **a** scanning electron microscopy (SEM) image, **b** adhesion assay. *B. subtilis*: **c** SEM image, **d** adhesion assay. *Chlorella*: **e** optical image, **f** adhesion assay. Reprinted from *Mater. Lett.* 2021, 285:129141. Copyright 2021 Elsevier

Bacterial adhesion to parylenes was tested with *E. coli* and *Bacillus subtilis* (*B. subtilis*) on pristine PDMS and PDMS coated with parylene C, parylene D [106], or parylene AF4 [107]. While bacterial adhesion to parylene D was only slightly reduced compared to PDMS, bacterial adhesion to parylene C or parylene AF4 was significantly reduced [106, 107]. Marine fouling on PDMS or PDMS coated with parylene AF4 was tested with the bacteria *Paracoccus pantotrophus* (*P*. *pantotrophus*) and *B. subtilis* as well as with algae *Chlorella*. Again, parylene AF4 showed excellent anti-adhesion and antifouling properties, particularly regarding *B. subtilis* and *Chlorella* (Fig. 6) [108].

Additionally, the material was biocompatible when tested with fibroblasts. The cell viability was almost 87% after a 24-h incubation time, suggesting that the toxicity towards these cells is negligible [107]. This could be improved by treating the parylene AF4 surfaces with oxygen to create a more hydrophilic surface. This treatment increased the viability of the fibroblasts to over 90%. Furthermore, the hemolysis rate of the plasma-treated parylene AF4 surfaces was less than 2%, which is below the medical safety threshold of 5%, confirming the biocompatibility of plasma-treated parylene AF4 [23].

Grids providing parylene areas separated by SiO_2_ were prepared with parylene N, C, D, and AF4 for culturing human astrocytes. The astrocytes preferred the parylene materials to locate. However, while all four parylenes attracted the cells, the cytoplasm of the astrocytes did not spread across the respective surfaces, showing an atypical morphology of the cells. The treatment of the grids with piranha solution (a mixture of H_2_O_2_ and H_2_SO_4_, both concentrated, in the ratio 5:3), a strong oxidizing agent, improved the cell morphology. On the treated grids, the astrocyte cytoplasm spread and was only limited by the respective parylene area. The cell morphology was now similar to that on polystyrene, the standard material for cell culture. Furthermore, all parylene materials supported Ca^2+^ signaling in the cells in a similar manner [29, 104]. A copolymer made from parylene C and parylene VT4 precursors resulted in a parylene that was more thermally stable than parylene C, while initial cell culture experiments showed similar biocompatibility [109]. Consequently, using a combination of expensive, heat-resistant parylene precursors and less expensive parylene C dimers to create copolymers could be a cost-effective method of producing parylenes that are more thermally stable than parylene C yet less expensive than pure, heat-resistant parylenes.

#### Heat-resistant parylenes in bioanalytical applications

Chips with silicon nitride apertures (diameter < 100 µm) were coated with parylene C or parylene AF4 to enable the formation of free-standing lipid bilayers (LBL). Since both parylenes are lipophilic, further precoating steps were unnecessary. Both parylenes enabled the formation of reproducible LBL within a few seconds. This includes at least six cycles of repetitive construction and destruction of the LBL. When pore-containing outer membrane protein F (OmpF) was incorporated into the LBL, the characteristic ion channel activities of the resulting bilayer lipid membrane (BLM), which comprises trimeric conductance steps, could be recorded [110]. Similar devices with free-standing BLM for electrophysiological measurements were developed that incorporated pore proteins directly or via outer membrane vesicles (OMVs) into the LBL. Parallel recordings of membrane transport processes were enabled by assembling several aperture-containing chips. Successful applications include the recording of fast gating events down to 1 ms and small amplitude gatings with current steps of around 3 pA. Parylene C and parylene AF4 were equivalent in their performance in this setup [111]. Both parylenes were also applied in the preparation of flexible multielectrode arrays based on iridium for electrical stimulation in prosthetics. Since the fabrication process involved iridium evaporation and patterning, parylene AF4 was a better choice than parylene C because of its higher thermal stability. Successful stimulation of the multielectrode array was demonstrated by the combination of the array with a retina isolated from a salamander and by implanting the array along the spinal cord of mice. Biostability was confirmed by 6-month retinal implantation in a canine eye [112].

The low autofluorescence of parylene AF4 was exploited in the development of a microelectrocorticography (microECoG) device based on parylene AF4 and indium tin oxide (ITO). The device enabled fluorescent two-photon imaging of Ca^2+^ signals of individual neurons as well as simultaneous 32-channel electrophysiology measurements to record the cortical electroencephalogram (EEG). This was demonstrated in vivo by implanting the device into mice that expressed a genetically encoded fluorescent calcium indicator [113]. The immunoresponse to the 32-channel microECoG device was investigated after implantation in mice up to 116 days. While an immunoresponse was evoked by the craniotomy window surgery, it was not enhanced by the parylene AF4. This makes parylene A4 an ideal choice for stable, biocompatible chronic implants [114].

**Fig. 7:**
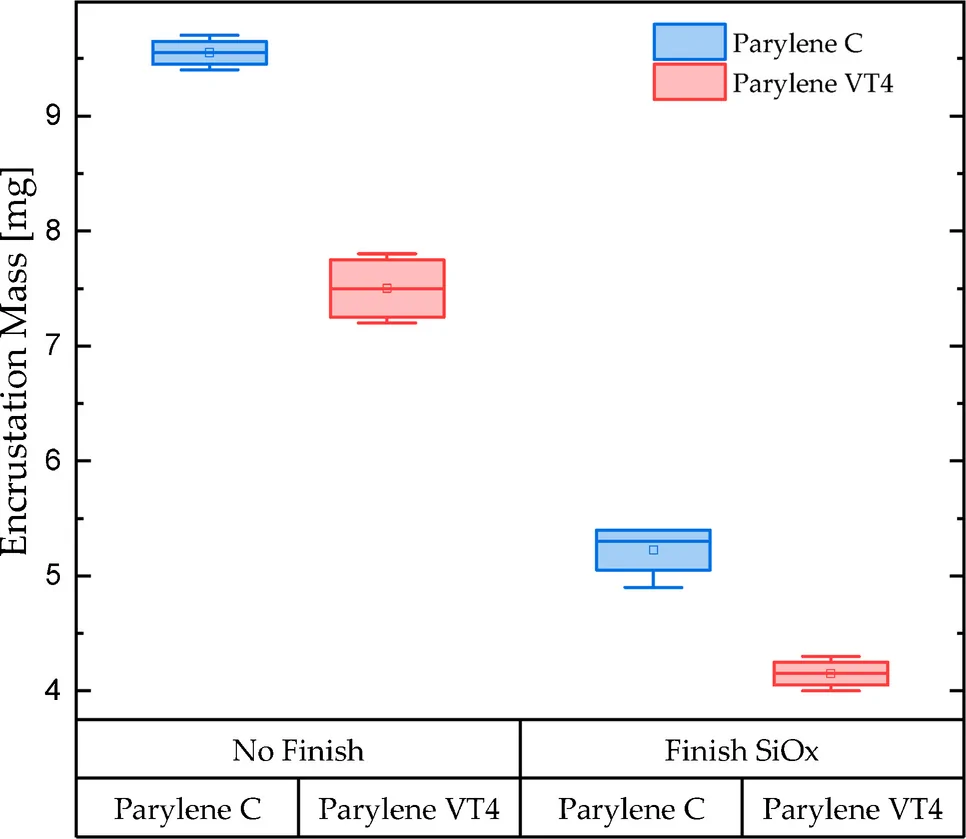
Urine encrustation on parylene C and on parylene VT4. The parylenes were used as is (“No Finish”) and with a SiO_x_ finishing coating (“Finish SiO_x_”). Reprinted from *Polymers* 2023, 15(17):3559. Published open access by MDPI under a CC BY license

Parylene C and parylene VT4 films were tested as coatings to prevent encrustation of a MEMS sensor system developed to measure urinary bladder pressure. The experiments were performed in synthetic urine. The parylenes were used as is or after receiving a finishing coating with SiO_x_. The coating reduced the contact angles from above 90° to below 60° for both parylenes, making the surfaces more hydrophilic. In both cases, the amount of encrustation on the parylene VT4-coated devices was lower than on the parylene C-coated devices, especially after the SiO_x_ finishing coating (Fig. 7) [115].

Furthermore, parylene VT4 was used as biocompatible and hermetic encapsulation of a piezoelectric micromachined ultrasonic transducer (PMUT). PMUT implants may serve as brain implants or charging applications for other implants. First studies investigated the acoustic output of PMUT arrays after encapsulation, while tests with cells or tissue are still pending [116].

### Parylenes for protein immobilization: parylene A, parylene AM, and parylene H

#### Parylenes for protein immobilization: overview

**Fig. 8:**
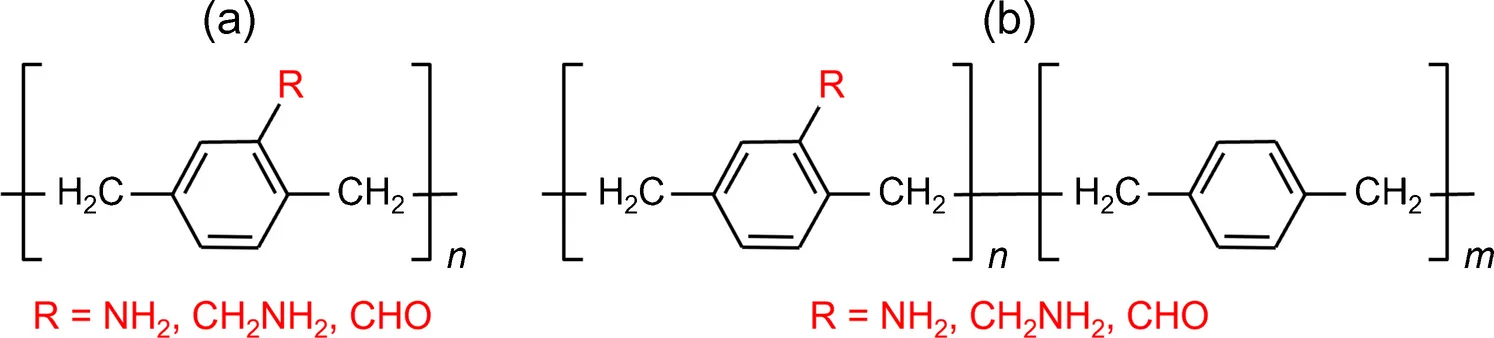
Molecular structures of **a** homopolymeric and **b** copolymeric parylenes providing amino groups (parylene A), aminomethyl groups (parylene AM) or formyl groups (parylene H)

Parylenes with functional groups facilitating the immobilization of proteins or peptides mainly include parylenes with amino groups (parylene A), aminomethyl groups (parylene AM), or formyl groups (parylene H). The basic molecular structures of these parylenes are shown in Fig. 8.

As depicted in Fig. 8, parylenes A, AM, and H have been introduced as homopolymers and copolymers with unmodified monomer units, i.e., the precursors used in the Gorham process have two or one functional group, respectively (Fig. 1). Both types of precursors are possible as demonstrated by the example of parylene A, i.e., steric hindrance is not an issue with the homopolymer [3]. The applications described below applied either homopolymer or copolymer of the respective parylene (Fig. 8). However, to the best of our knowledge, a direct comparison between homopolymeric and copolymeric parylenes that provide the same functional group has not been made regarding their performance in bio-applications. Therefore, in the following, the terms “parylene A,” “parylene AM,” and “parylene H” refer to both homopolymer and copolymer, emphasizing the functional group as the key element of the parylene properties.

Parylene A and parylene AM, providing amino groups and aminomethyl groups, respectively, allow coupling with amino groups of proteins or peptides by using dicarboxylic acid anhydrides or dialdehydes as cross-linkers forming peptide bonds or Schiff bases, respectively. Similar to the latter, the coupling of parylene H providing formyl groups to amino groups of proteins or peptides results in Schiff base formation. However, an additional linker reagent is not required. In the following applications, parylene A, parylene AM, and parylene H were used with and without further functionalization.

#### Parylenes for protein immobilization: use in cell culture and cell-sorting

Culture dishes made of polystyrene—the standard material for cell cultivation—were coated with parylene A, parylene AM, or parylene H to test the impact of the microenvironment created by the parylenes’ functional groups on cell cultures.

PC-12 cells, which exhibit a neuron-like morphology, showed low adhesion to parylene C and parylene A. However, they adhered to parylene AM and parylene H in a manner similar to polystyrene [117]. Furthermore, PC-12 cells differentiated on parylene AM and parylene H, as evidenced by the neurite overgrowth. The cellular differentiation of neurite overgrowth was even larger with parylene H than with polystyrene [118]. U-937 monocytes, which do not typically adhere to surfaces such as polystyrene, were found to adhere to amine-rich polymers, such as parylene AM [119, 120]. MG-63 osteoblast-like cells were found to adhere more to parylene A than to polystyrene, but not to parylene N. UV exposure increased adhesion to both parylene A and, particularly, parylene N, with adhesion to the latter now exceeding that to polystyrene. The introduction of functional groups through UV treatment increased the hydrophilicity of the materials. This was particularly impactful for parylene N, which initially lacked functional groups (Fig. 2a). MG-63 cell proliferation rates on parylene A with or without UV treatment, as well as on UV-treated parylene N, were similar and significantly higher than those on untreated parylene N and on polystyrene [121].

**Fig. 9:**
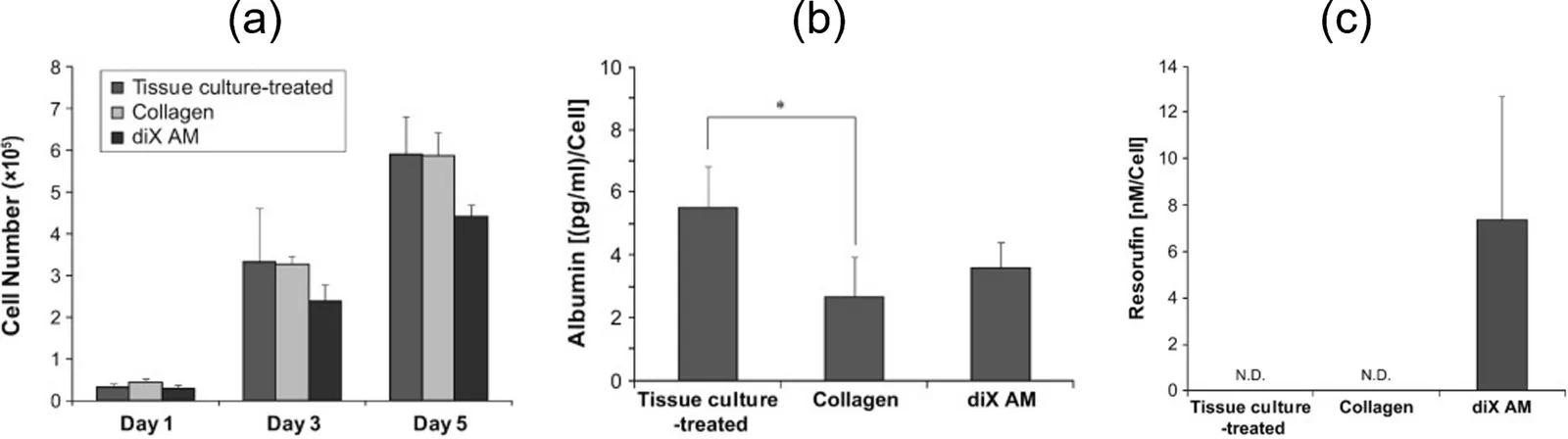
HepG2 cells cultured on plates treated with tissue culture or coated with collagen or parylene AM. **a** HepG2 cell proliferation. **b** Albumin secreted in HepG2 cells as determined by enzyme-linked immunosorbent assay (ELISA). **c** Enzymatic activity of CYP1 in HepG2 cells as determined by an EROD assay. Data represent three (**a**) or four (**b**, **c**) independent experiments. Reprinted from *Mater. Sci. Eng. C-Mater. Biol. Appl.* 2015, 46:190-4. Copyright 2015 Elsevier

Hepatocellular carcinoma (HepG2) cells were cultivated on tissue culture-treated plates as well as on plates coated with collagen or parylene AM (Fig. 9). The cell numbers were slightly lower on parylene AM than on collagen and tissue culture-treated plates, which were similar. However, the morphology and albumin secretion of cells on parylene AM and collagen were similar, though albumin secretion was slightly downregulated compared to cells on tissue culture-treated plates. The enzymatic activity of CYP1, a family of cytochrome P450 (CYP) enzymes, was only detected on HepG2 cells cultured on parylene AM. Gene expression analysis confirmed this finding by showing induced expression of the respective gene in cells cultured on parylene AM without an additional factor. This finding suggests that parylene AM is a promising cell culture material that offers specialized functions [122].

Antibody-producing hybridoma cells were adhered to parylene AM, parylene H, parylene N, parylene C, and polystyrene [123]. Both parylene N and parylene C were tested with and without UV treatment. As observed before with MG-63 cells [38, 121], UV exposure improved cell adhesion and proliferation on parylene C and parylene N by introducing oxygen-containing functional groups. After 72 h, significantly more cells adhered to the parylene surfaces than to the polystyrene surfaces, with the exception of the untreated parylene N surface. The enhanced cell proliferation corresponded to an increased antibody production. Therefore, except for untreated parylene N, more antibody was obtained with the parylenes than with polystyrene, confirming the benefit of parylenes with oxygen- or nitrogen-containing functional groups for cell culture [123].

Parylene AM was used to coat the microchannels within an adhesion-based cell sorter. In one section of the cell sorter, the amino groups were functionalized with a biotin-NHS ester, streptavidin, and biotin-labeled anti-CD31 or anti-CD86 antibody. HUVECs highly express CD31. In the cell sorter, HUVECs are slowed down in the section coated with anti-CD31, but their velocity remains unaffected in the section coated with anti-CD86. Furthermore, the cell sorter could separate HUVECs from human leukocytes when they were provided in a mixture. The parylene AM coating facilitated antibody immobilization in the channel, resulting in a successful cell sorter design [124, 125].

#### Parylenes for protein immobilization: use in microplates for immunoassays

Microplates used for immunoassays are commonly made of polystyrene. Since polystyrene does not provide functional groups for chemical coupling, capture molecules are immobilized by adsorption. Microplates were coated with parylene A, parylene AM, or parylene H, which provide functional groups for protein coupling, to investigate the performance of covalently coupled proteins and peptides in immunoassays.

Adsorption may not be effective when the capture molecules are small proteins or peptides. Therefore, the immobilization of two proteins (human chorionic gonadotropin (hCG) and horseradish peroxidase (HRP)) and a 15-mer peptide (cyclic citrullinated peptide (CCP)) was investigated with polystyrene, protein A, and protein H. A significantly higher immobilization efficiency was obtained by covalent immobilization on parylene A or H than by adsorption on polystyrene for both the proteins and the peptide. Furthermore, parylene H produced a superior immobilization efficiency compared to parylene A due to its simpler one-step procedure and significantly higher signal [126]. This was exploited in an assay to detect anti-CCP antibodies, which are found in the serum of rheumatoid arthritis (RA) patients. Microplates were coated with parylene H, and then CCP was immobilized. Anti-CCP was detected using a sandwich assay format with fluorescent-labeled secondary antibodies that bound to the anti-CCP. This assay was comparable to a standard enzyme-linked immunosorbent assay (ELISA) in its ability to identify RA patient samples as positive and healthy control samples as negative. In other words, it had similar diagnostic sensitivity and specificity [127]. Site-directed immobilization of antibodies onto parylene H was performed to ensure the accessibility of the antibodies’ binding sites (i.e., the F_ab_ fragments) after immobilization. First, poly-L-lysine was covalently coupled to the parylene H-coated microplate, resulting in a positively charged surface. This allowed for the immobilization of *E. coli* via charge interactions due to the negatively charged outer membrane of the cells [128]. The *E. coli* used here autodisplayed Z-domains, an engineered protein A domain that binds to the F_c_ fragment of antibodies while leaving the F_ab_ fragments accessible [129]. Anti-CRP antibodies were immobilized to the Z-domains and, for reference, adsorbed onto polystyrene. In a colorimetric sandwich ELISA for detecting CRP, the assay signals obtained using the orientation-controlled antibodies were more than 20 times higher than those obtained using adsorbed anti-CRP antibodies [128].

Parylene A-coated microplates were used in immunoassays to detect substance P (SubP), a neuropeptide and neurotransmitter being discussed as a potential diagnostic marker for acute myocardial infarction (AMI). To meet the high requirements for detecting low concentrations in the range of 1–100 pg/mL in healthy controls, antibodies were labeled with silica spheres encapsulating a quantum dot layer (SQS), which is highly photoluminescent. These SQS-labeled antibodies replaced the enzyme-labeled antibodies used in ELISA, resulting in SQSLISA. Using the photoluminescent sandwich SQSLISA, results were twice as high when the capture antibody was covalently immobilized on the parylene A-coated polystyrene microplate than when it was adsorbed directly onto the polystyrene. Competitive assays were performed by covalently immobilizing SubP on parylene A-coated microplates. The SQS-labeled antibodies were mixed with SubP standard or samples. After incubation, any remaining antibodies with free binding sites bound to the SubP-modified microplate, and photoluminescence was measured. The dynamic range of the competitive SQSLISA was determined to be 1–10^4^ pg/mL. Excellent diagnostic sensitivity and selectivity were obtained with clinical AMI and healthy control samples, exceeding the results obtained with a commercial competitive ELISA, which had a dynamic range of 30–10^4^ pg/mL [130].

**Fig. 10:**
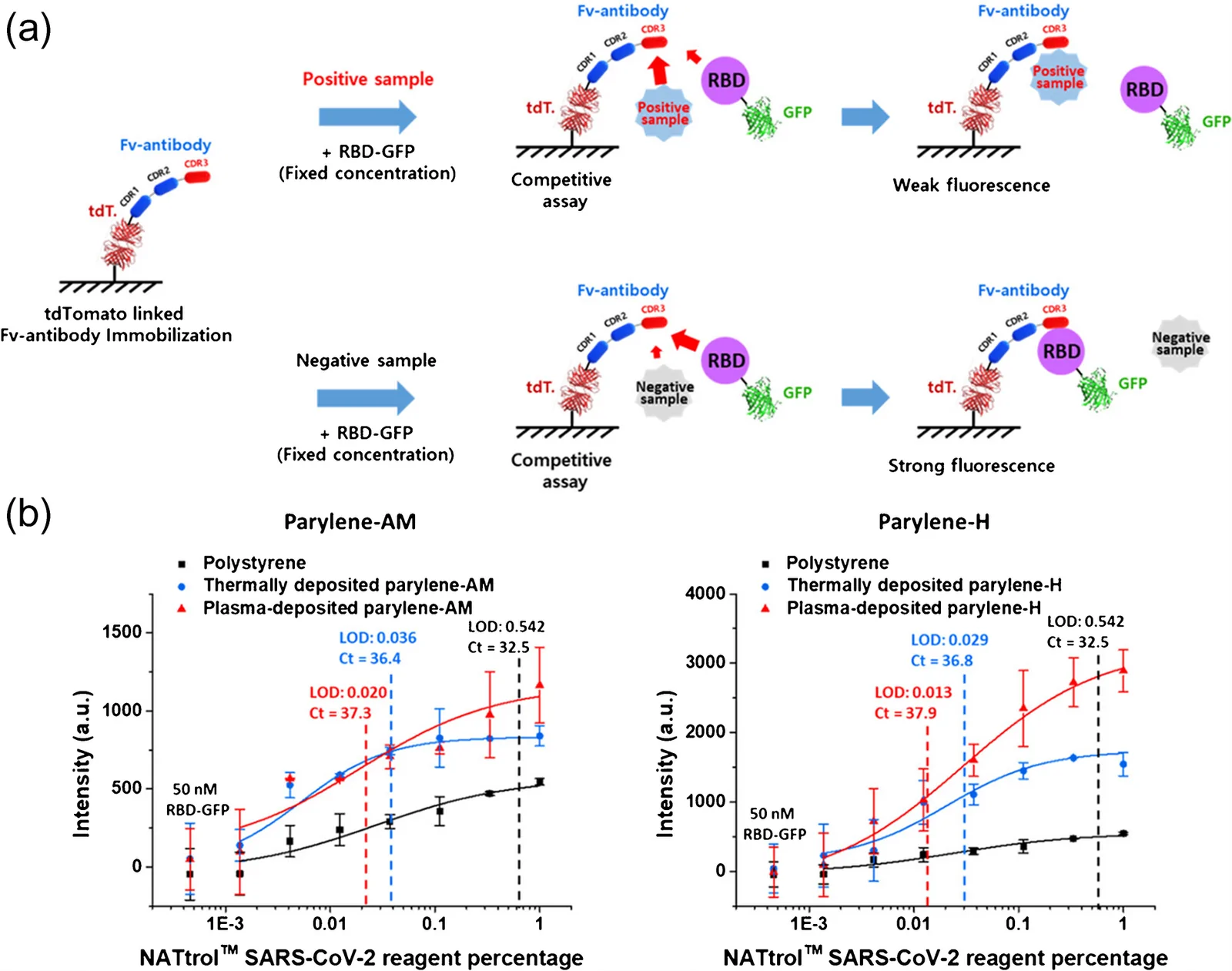
Competitive immunoassay for the detection of SARS-CoV-2 using the RBD of the SARS-CoV-2 spike protein. Anti-RBD Fv antibodies labeled with tdTomato were adsorbed onto polystyrene or covalently immobilized onto either thermally or plasma-deposited parylene AM or parylene H. **a** Schematic of the competitive immunoassay with GFP-labeled RBD in standard solution and RBD from SARS-CoV-2 in sample competing for the antibody binding sites. **b** Fluorescence signals obtained for the detection of SARS-CoV-2 in real samples by competitive immunoassay. Reprinted from *ACS Appl. Mater. Interfaces* 2024, 16(46):63255-67. Copyright 2024 American Chemical Society

In a recent study, plasma decomposition of the sublimated precursors within the Gorham process for parylene deposition (Fig. 1) was investigated as an alternative to conventional thermal pyrolysis. Parylene H and parylene AM films based on plasma decomposition exhibited lower water contact angles and higher immobilization efficiencies than the corresponding films based on pyrolysis. The respective functional groups remained on the plasma-deposited parylene surfaces and were partly complemented by oxygen- and nitrogen-containing species, which explains the lower contact angles. HRP, green-fluorescent protein (GFP), or Fv-antibodies labeled with tdTomato, a fluorescent protein, were covalently immobilized on microplates coated with parylene AM or parylene H and adsorbed onto polystyrene [10]. As before [126, 130], covalent immobilization yielded a significantly higher immobilization efficiency than adsorption of the proteins. Additionally, the immobilization efficiency was further enhanced by using plasma-deposited parylenes. The best results were obtained with plasma-deposited parylene H. Competitive immunoassays were performed with immobilized tdTomato-labeled Fv-antibodies against the receptor-binding domain (RBD) of the SARS-CoV-2 spike protein. GFP-labeled RBDs in standard and SARS-CoV-2 in samples competed for the antibodies’ binding sites on the surface. In accordance with the results obtained from the immobilization efficiency experiments, the best assay results were obtained with plasma-deposited parylenes (Fig. 10). The low limit of detection (LOD) values were close to the requirements for the detection of SARS-CoV-2 to diagnose COVID-19 [10].

#### Parylenes for protein immobilization: use in biosensors

Parylene A, parylene AM, and parylene H were investigated as linker layers for further immobilization steps in biosensor transducers using optical or electrochemical detection. Furthermore, the conformal coating properties of parylenes are particularly suitable for biosensor setups that use nanowires or porous membranes.

SPR gold chips were coated with parylene A or parylene H. HRP was covalently immobilized on the parylenes and adsorbed onto pure gold chips for reference. SPR measurements revealed that the LOD values obtained with parylene A and parylene H were improved more than tenfold and 1000-fold, respectively, compared to direct adsorption on gold chips [126, 131]. These results confirmed those obtained with parylene-coated microplates, as described in the previous section. Site-directed immobilization of anti-immunoglobulin G (IgG) antibodies onto parylene H-coated SPR gold chips was achieved through the covalent binding of protein G, which binds to the F_c_ fragment of antibodies in a manner similar to protein A. Detection of the corresponding IgG produced higher signals when anti-IgG was coupled via protein G than when it was covalently immobilized onto parylene H because the former method ensures accessible binding sites [132]. Furthermore, similar as described for polystyrene microplates in the section before [128], parylene H-coated SPR gold chips were coated with poly-L-lysine and *E. coli* by charge interactions between the positively charged poly-L-lysine surface and the negatively charged outer membrane of the *E. coli* cells. The *E. coli* used here autodisplayed Z-domains, which were used for the site-directed immobilization of anti-CRP to detect CRP. Other approaches involved adhering the *E. coli* cells to pure SPR gold chips or immobilizing the cells by charge interactions on poly-L-lysine, which was itself immobilized by charge interactions on the negatively charged gold surface of the SPR chips. SPR measurements for the detection of CRP yielded the best results when *E. coli* cells were immobilized via poly-L-lysine covalently immobilized on parylene H. The LOD was 1 ng/mL, which is much lower than the cut-off value of 1 µg/mL for generally inflammatory diseases; however, measurements in real samples are still pending [133].

A cadmium sulfide (CdS) nanowire photosensor was developed for the detection of *E. coli* based on the fact that its outer membrane contains lipopolysaccharides (LPS). The photosensor was coated with parylene C and photoresist as protective layers and with parylene H as a linker layer for subsequent immobilization. The Z-domains of protein A were covalently immobilized on parylene H, which enabled site-directed binding of anti-LPS antibodies. After incubation of *E. coli* samples, human anti-LPS was incubated as a second antibody, followed by anti-human IgG labeled with HRP as a third antibody. Addition of luminol and hydrogen peroxide produced a chemiluminescence signal that was measured with the CdS nanowire photosensor. The LOD was estimated to be lower than 10^4^ *E. coli*/mL [134].

**Fig. 11:**
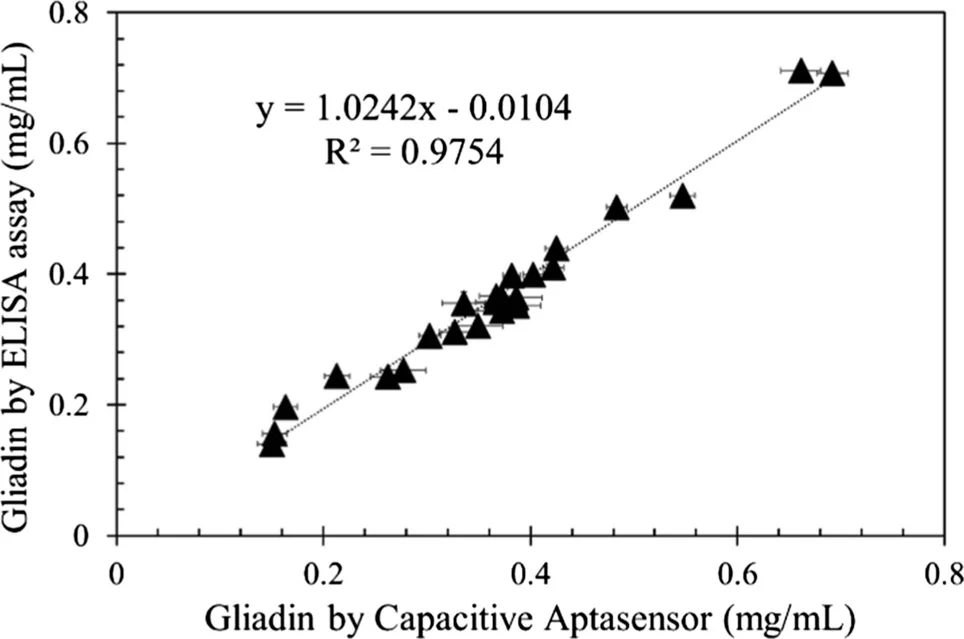
Detection of gliadin in 24 commercial wheat flour samples. Correlation between results obtained from capacitive aptasensor measurements (*n* = 3) and ELISA tests (*n* = 3). Reprinted from *ACS Sens.* 2024, 9(7):3689-96. Copyright 2024 American Chemical Society

An interdigitated electrode (IDE) capacitive biosensor equipped with nanoislands was coated with parylene A, taking advantage of the conformal coating properties of parylenes. In a preliminary experiment, HRP was adsorbed onto IDEs with and without nanoislands, as well as covalently immobilized onto the IDE with nanoislands coated with parylene A. The latter immobilization method resulted in the highest signal in subsequent measurements with anti-HRP. Therefore, HBsAg was immobilized on the parylene A-coated IDE with nanoislands to detect anti-HBsAg in spiked serum. The sensor was selective for the corresponding antibody at concentrations as low as 1 ng/mL. Signals obtained for anti-CRP and anti-IgG were significantly lower, which confirms the potential of the parylene A-coated IDE with nanoislands as a capacitive biosensor in the field of diagnostics [135]. Another capacitive biosensor was developed using a screen-printed carbon electrode as the transducer for capacitive aptasensing of gliadin. Gliadin, a protein component of gluten, is considered the primary trigger of the autoimmune response in celiac disease. The electrode was first coated with a parylene C layer for insulation, followed by a parylene AM layer for protein immobilization. An aptamer directed against gliadin was labeled with an amino group and covalently immobilized on the parylene AM layer. Measurements using extraction mixtures from commercial wheat flour samples revealed an excellent linear correlation between the results obtained with the capacitive aptasensor and those obtained with a commercial ELISA (Fig. 11). Furthermore, the LOD for gluten was determined to be 14 ppm, which is below the 20 ppm threshold specified by European and US legislation. This makes the capacitive aptasensor with parylene AM linking layer a promising tool for fast and reliable gluten detection [136].

A chronoamperometric enzyme sensor for urea detection was developed by assembling a screen-printed electrode setup with a porous polytetrafluoroethylene (PTFE) membrane that was coated with parylene A and covalently immobilized urease in a PDMS flow cell. The biosensor is based on the enzymatic hydrolysis of urea into ammonia and carbon dioxide. The waste peritoneal dialysate of a patient with chronic renal failure was analyzed for urea concentrations at a flow rate of 0.5 mL/min. The LOD for urea was determined to be below 1.2 mM, which was the lowest concentration in the dialysate. The biosensor was considered suitable for monitoring urea concentrations of up to 20 mM, a level that would be appropriate for clinical applications, such as portable dialysis systems [137].

#### Parylenes for protein immobilization: use in MALDI-TOF MS

A thin layer of parylene H was used to coat the target plate for MALDI-TOF MS. This enabled the covalent immobilization of capture molecules on the parylene H coating for subsequent measurements of biomolecular interaction analysis. Streptavidin was used as a model capture molecule, and biotin-labeled CCP peptide was used as the ligand. When streptavidin was adsorbed onto a standard matrix plate, the streptavidin peak was clearly visible, while the ligand sample peaks were barely discernible. However, when streptavidin was covalently immobilized on the protein H-coated matrix plate, the streptavidin peak was suppressed, and the ligand sample peaks were clearly visible with an estimated LOD of 1 ng/mL. Thus, the parylene H linking layer enabled covalent coupling of capture molecules on the MALDI target plate. This reduced the matrix effects in the subsequent measurement and allowed the investigation of biomolecular interactions with MALDI-TOF [138].

### Parylenes for click chemistry: ethynyl- and maleimide-functionalized parylenes

#### Parylenes for click chemistry: principle

The most commonly used parylene compounds that allow for click chemistry [139] are parylenes with ethynyl or maleimide groups. These parylenes have only been presented as copolymers with unmodified monomer units. It was shown that CVD of precursors with two ethynyl groups did not result in functional homopolymeric parylene layers, as would have been expected according to the Gorham process (Fig. 1, left path). Instead, side products were formed. However, CVD of the precursor with one ethynyl group resulted in the desired copolymer (Fig. 1, right path) [140]. Similar results may have been obtained with other clickable parylenes, so that parylene with maleimide groups was directly introduced with a monofunctional precursor [141].

Parylene with ethynyl groups undergoes click reactions with azide (N_3_) groups in the presence of copper(I) as a catalyst. Parylene with maleimide groups undergoes click reactions with thiol (SH) groups when initiated by heat, light, or radicals. Though these reactions are promising due to their speed, simplicity, and high efficiency [139], the underlying parylenes are still rarely used in bioanalytical applications. One reason may be their limited availability, as they are not as widely available as other parylenes (Table 1).

Parylenes suitable for click chemistry can have one or more types of functional groups. These parylenes have primarily been used to create patterned or structured surfaces, e.g., for spatially controlled cell cultivation. Examples are shown in the following sections.

#### Parylenes for click chemistry with one type of functional group

**Fig. 12:**
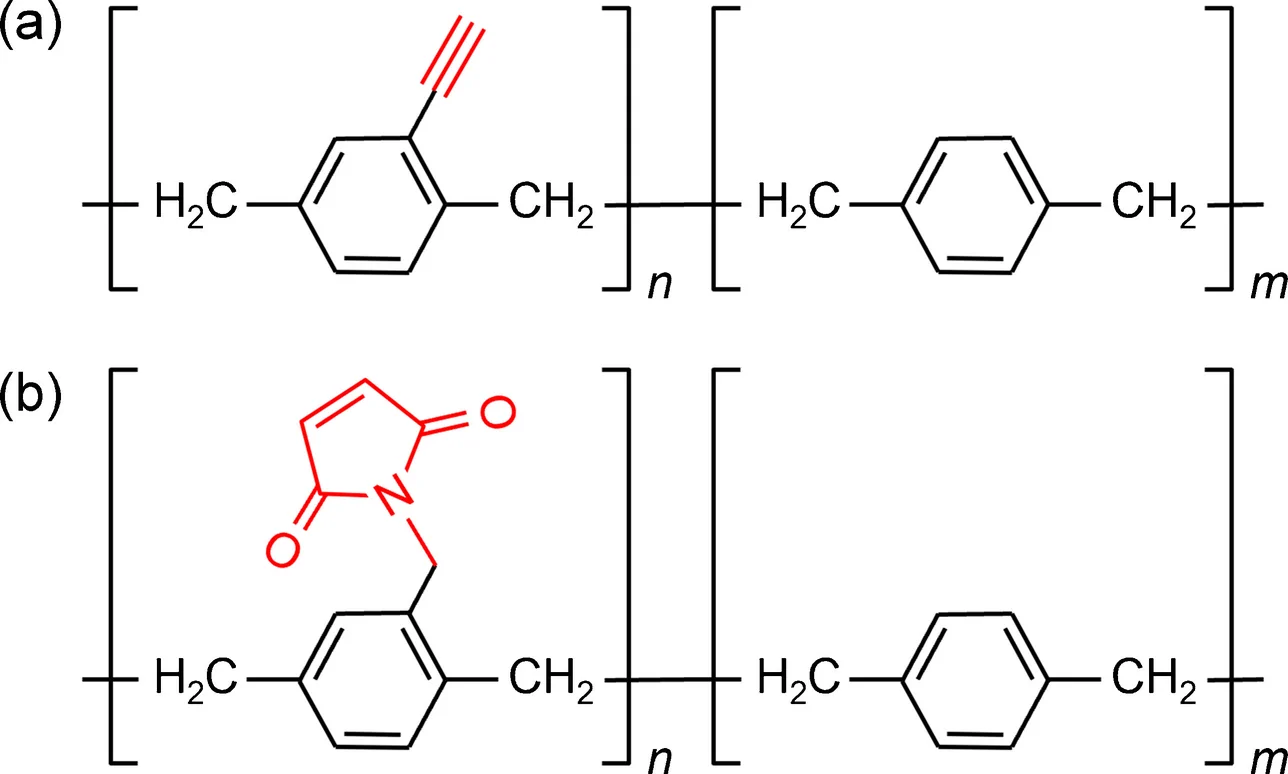
Molecular structures of parylenes for click chemistry: **a** poly(4-ethynyl-*p*-xylylene-*co*-*p*-xylylene (parylene X) and **b** poly(4-N-maleimidomethyl-*p*-xylylene-*co*-*p*-xylylene)

The most common parylenes for click chemistry providing one type of functional group are shown in Fig. 12. Parylene with ethynyl groups was introduced as parylene X [142].

**Fig. 13:**
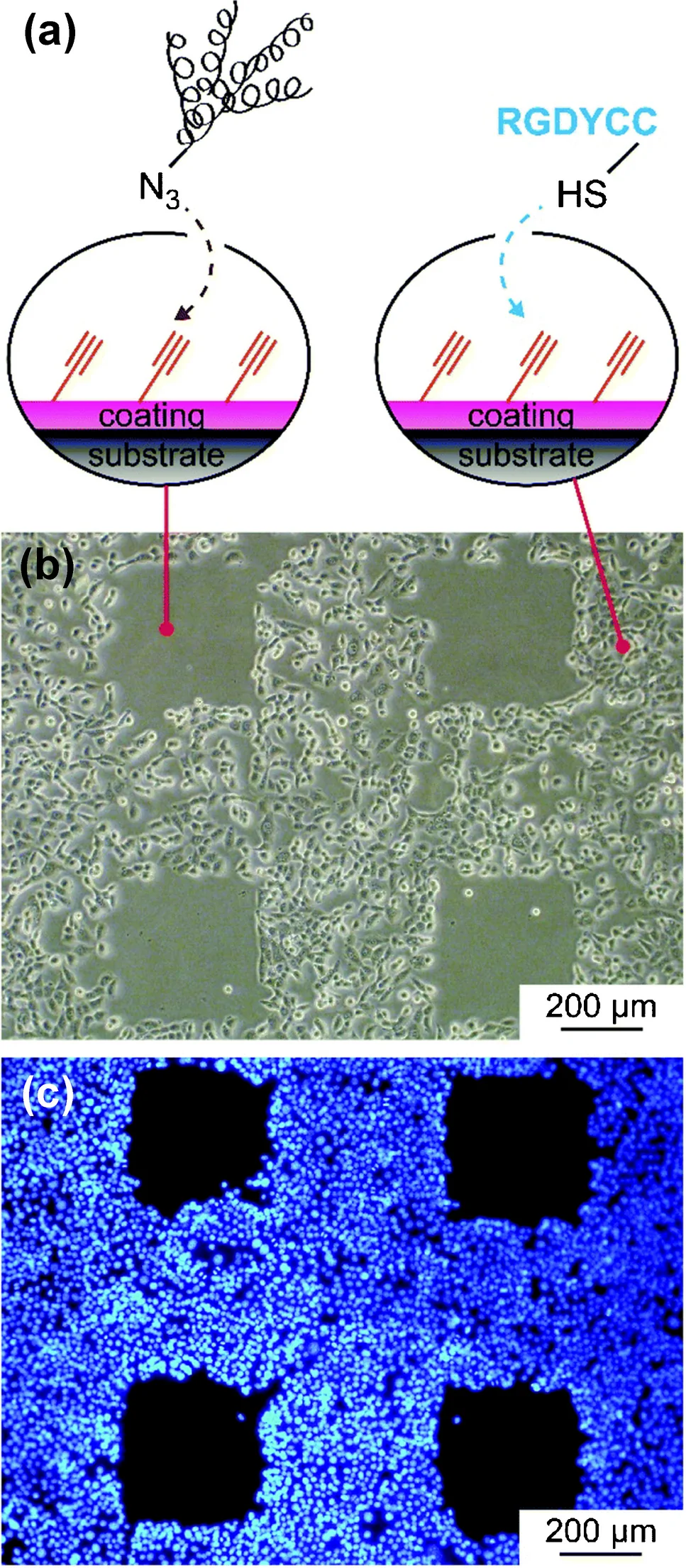
Fibroblasts cultured on parylene X functionalized subsequently with azido-labeled PEG by microcontact printing and with a thiol-terminated RGD peptide using photochemistry and a photomask. **a** Overview of the reactions with the ethynyl groups. **b** and **c** Microscopic and DAPI-stained images, respectively, after 24 h of fibroblast cell growth. Reprinted from *Biomater. Sci.* 2016, 4(2):265-71. Copyright 2016 Royal Society of Chemistry

Parylene X-coated substrates were treated with azide-containing biotin-based ligands to make the ethynyl groups visible by immobilizing fluorescent-labeled streptavidin. Patterns were obtained on the surface by microcontact printing of the copper(I) catalyst or by dip-pen nanolithography (DPN) writing of biotin-labeled azide molecules. Fluorescence micrographs confirmed the patterns [140, 143]. Furthermore, parylene X was patterned by microcontact printing of azido-labeled PEG, followed by a photochemically activated thiol–yne click reaction with a thiol-terminated RGD peptide using a photomask. As expected, fibroblasts adhered selectively to the peptide-functionalized areas while being repelled by the PEG (Fig. 13) [144].

**Fig. 14:**
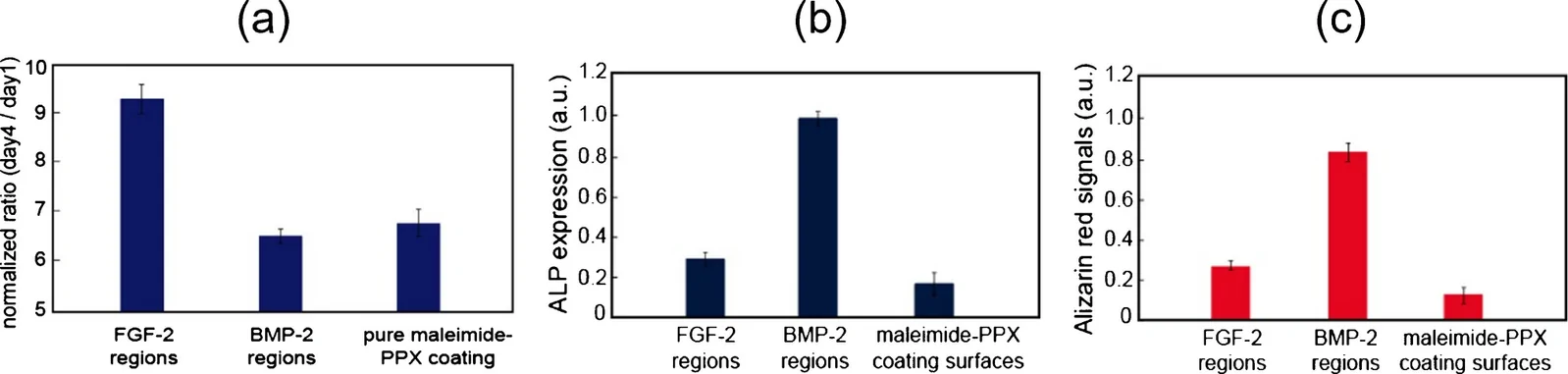
MC3T3-E1 cell proliferation and differentiation on substrates coated with parylene providing maleimide groups, as well as on substrates with subsequent coatings of FGF-2 or BMP-2 (*n* = 3). **a** MC3T3-E1 cell proliferation. **b** ALP expressed by MC3T3-E1 cells. **c** Calcium deposition, visualized by Alizarin red staining. Reprinted from *Langmuir* 2017, 33(36):8943-9. Copyright 2017 American Chemical Society

Substrates coated with parylene containing maleimide groups were patterned using microcontact printing. Stamping with thiolated fluorescent dyes made the grid-like structure visible. Stamping with thiol-labeled PEG and subsequent application of fluorescent-labeled fibrinogen produced a reverse pattern because the antifouling properties of the PEG repelled the protein, causing it to adsorb on the unmodified area of the parylene-coated substrate. A similar method was used to pattern cells on substrates coated with parylene carrying maleimide groups. First, thiol-PEG was stamped on the surface. Then, a cell-adhesive peptide containing a terminal thiol group was applied, which bound to the remaining unmodified areas. Subsequently, bovine aorta endothelial cells (BAECs) adhered to the peptide-modified areas on the substrate, but not to the PEG-modified areas [141]. Similar results of controlled cell attachment were obtained with murine preosteoblasts (MC3T3-E1). This was further developed by patterning different proteins to initiate spatially controlled cell differentiation on a single substrate. Fibroblast growth factor 2 (FGF-2), which promotes cell proliferation, and bone morphogenetic protein (BMP-2), which initiates osteogenesis, were subsequently applied to the parylene-coated substrate with maleimide groups in a defined pattern. Preosteoblast proliferation on the FGF-2-coated areas was confirmed by increased cell numbers compared to BMP-2-coated areas, which exhibited the same number of cells as unmodified areas. Osteogenesis on the BMP-2-coated areas was confirmed by enhanced alkaline phosphatase (ALP) expression and increased calcium deposition compared to the FGF-2-coated or unmodified parylene-coated substrate areas (Fig. 14). Consequently, patterning different proteins makes it relatively easy to initiate various cell activities on the same substrate [145].

A parylene with a bifunctional group, 2-nitro-5-(prop-2-yn-1-yloxy)methylbenzyl carbamate, was introduced, that enabled both click chemistry and photochemical cleavage to reveal amino groups. Patterning was achieved through UV exposure via a photomask instead of microcontact printing. The feasibility of creating patterns with these functionalities was demonstrated by the reaction of the alkynyl groups with azido-labeled PEG-biotin, followed by UV exposure via photomask and reaction of the revealed amino groups with a fluorescent-labeled NHS-ester. A pattern for cell cultivation was formed using azido-labeled PEG for hydrophilic, cell-repellent areas and a benzophenone-labeled peptide that was coupled via photochemical reaction to the revealed amino groups. As expected, fibroblasts adhered preferentially to the peptide-coated areas [146].

Furthermore, a parylene surface providing different functional groups was obtained by sequential deposition of parylenes in a vapor-assisted micropatterning in replica structures (VAMPIR) process. First, the substrate was completely coated with parylene H, which provided formyl groups. Then, the surface was covered with a stencil, and parylene X, which provided ethynyl groups, was deposited on the parylene H surface where it was not covered by the stencil, resulting in a parylene X pattern. The functionality of the ethynyl groups was confirmed by reaction with azido-functionalized saccharides or peptide and subsequent binding of the corresponding fluorescent-labeled lectins or adhesion of HUVECs, respectively. The latter could be suppressed by immobilizing azido-functionalized PEG instead of peptide. Furthermore, a dihydrazide linker was used to immobilize mannobiose to the aldehyde groups, and azido-functionalized galactose was immobilized to the ethynyl groups. Reaction with the corresponding fluorescent-labeled lectins concanavalin A and peanut agglutinin (PNA), respectively, allowed the characterization of the spatial resolution of the two saccharides patterned on the surface [147].

#### Parylenes for click chemistry with two types of functional groups

**Fig. 15:**
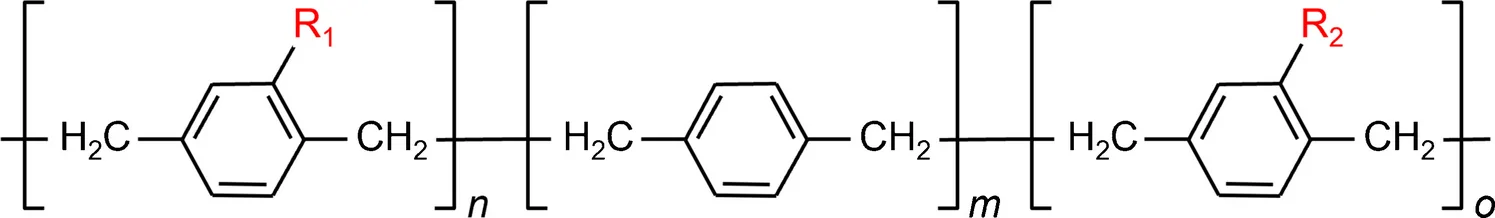
Parylene copolymer with two different functional groups

A parylene surface with different functional groups can be obtained through the sequential deposition of different parylenes using a stencil (VAMPIR process) [147], as shown above. Another possibility is the simultaneous deposition of precursors with different functional groups (i.e., CVD copolymerization), which results in parylene copolymers with two or more types of functional groups (Fig. 15). Typically, at least one of these types of functional groups is clickable.

Microcontact printing was used to create the patterns on the following bifunctional parylene surfaces. A bifunctional parylene surface was designed comprising ethynyl groups and formyl groups. The ethynyl groups were functionalized with an azido-labeled peptide. The formyl groups were functionalized with heparin via a dihydrazide linker. Binding reactions with anti-heparin and FGF-2 (here called basic fibroblast growth factor (bFGF)) demonstrated both the accessibility of the heparin epitopes to the corresponding antibody binding sites and the biological activity of the heparin [148]. Another bifunctional parylene surface comprised ethynyl groups and pentafluorophenyl ester groups. The ethynyl groups were functionalized with an azido-labeled cyclic RGD peptide. The surface was also functionalized with epidermal growth factor (EGF), which was immobilized through reaction with the ester group. HUVECs adhered best to the peptide-coated areas, regardless of whether EGF was immobilized or not. The EGF induced phosphorylation of the EGF receptor expressed by A431 cells, which are derived from a human epidermal carcinoma cell line. This confirms the biological activity of the immobilized EGF [149]. Furthermore, a double-clickable parylene was introduced exhibiting ethynyl groups and maleimidomethyl groups. The ethynyl groups were functionalized with azido-functionalized PEG, and the maleimide groups were functionalized with thiol-conjugated biotin. Subsequent binding of a fluorescent-labeled streptavidin was confined to the biotin-functionalized areas, leaving the PEG areas free [144].

Finally, a bifunctional parylene was created that provided aminomethyl and ethynyl groups for cultivating hematopoietic stem and progenitor cells (HSPCs). The cellular Notch ligand Delta-like 1 (DLL1), a membrane protein, was coupled to the amino groups via its carboxyl group and EDC/NHS activation. An azido-labeled RGD peptide was immobilized by reacting with the ethynyl groups. Microcontact printing was not used here. Rather, the ratio of parylene precursors in the CVD copolymerization process was adjusted to 5:1 (aminomethyl:ethynyl groups) to optimize HSPC proliferation. This approach may be an easier way to optimize cell cultivation than other approaches using bifunctional parylenes because it does not require surface patterning [150].

## Summary and conclusion

Parylene polymers are a highly versatile class of polymers that combine conformal deposition, excellent barrier properties, and biocompatibility. Therefore, they are increasingly recognized as not only protective and insulating coatings, but also a versatile platform of materials for bioanalytical devices and cell culture systems. Parylene C and parylene N are the most widely used and extensively studied polymers in this class. They are widely available, and their deposition parameters are well established. Despite their chemical inertness, they continue to provide valuable insights into fundamental processes such as protein adsorption and cell-surface interactions. Nevertheless, a wide range of post-deposition modification strategies has been developed, including physical treatments and chemical reactions. These surface modifications considerably expand the range of applications to include specific bioanalytical applications, such as biosensors.

In addition to common parylenes, heat-resistant parylenes, such as parylene D, AF4, and VT4, offer new possibilities for devices requiring elevated temperatures or UV exposure. However, their role in direct biofunctionalization is less prominent, as many applications rely on their intrinsic properties rather than chemical modification. In contrast, parylenes with amino or formyl groups have clear advantages in biointerface engineering. They enable the efficient covalent immobilization of biomolecules according to standard protocols, creating application-specific biointerfaces. These materials have demonstrated their value in immunoassays, enzyme assays, and biosensors. At the same time, these functionalized surfaces can directly influence cellular responses, showing that the chemical microenvironment is as important as subsequent functionalization. Additionally, parylenes with click-reactive groups permit comparatively easy, spatially resolved surface patterning, as demonstrated in advanced cell culture concepts. Their ability to create complex, application-specific architectures is particularly relevant for next-generation biosensing and tissue engineering approaches.

Apart from the parylenes discussed here, there are more parylenes, or at least their precursors, available. Research balances between developing precursors, which require determining the deposition parameters, and functionalizing known parylenes, for which the deposition parameters are already known. However, many of these precursors and recent methods of functionalizing parylene C or N have yet to be adopted for bioanalytical applications, despite their significant potential. Additionally, the copolymerization of available parylenes offers considerable potential because it may enable the combination of complementary properties, such as thermal stability and biocompatibility, within a single coating system.

Despite the considerable progress that has been made, several challenges remain. These include the limited commercial availability of specialized parylene precursors compared to standard commercial materials; the need for optimized, standardized deposition processes for functionalized variants; and the limited number of systematic, comparative studies across different parylene types and deposition parameters. Further advances are also expected from improved post-deposition modification methods for widely available parylenes, which could provide a practical alternative to synthesizing specialized precursors. Additionally, future work should consider practical factors such as cost, long-term coating performance, and possible environmental concerns associated with some fluorinated derivatives. Future progress will likely depend on improved access to functional precursors, further investigation of copolymerization and surface-modification strategies, and more systematic studies that directly compare the properties and application performance of different parylene types. These efforts are essential for fully exploiting the potential of parylenes in next-generation biosensors, biomedical coatings, and engineered cell culture systems.

## Data Availability

Data sharing is not applicable to this article as no datasets were generated or analyzed during the current study.

## References

[1] Hassan Z, Varadharajan D, Zippel C, Begum S, Lahann J, Bräse S. Design strategies for structurally controlled polymer surfaces via cyclophane-based CVD polymerization and post-CVD fabrication. Adv Mater. 2022;34(37):2201761. 10.1002/adma.202201761. 35555829

[2] Gorham WF. A new, general synthetic method for the preparation of linear poly-p-xylylenes. J Polym Sci A Polym Chem. 1966;4(12):3027–39. 10.1002/pol.1966.150041209.

[3] Lahann J, Langer R. Novel poly(p-xylylenes): thin films with tailored chemical and optical properties. Macromolecules. 2002;35(11):4380–6. 10.1021/ma011769e.

[4] Mitu B, Bauer-Gogonea S, Leonhartsberger H, Lindner M, Bauer S, Dinescu G. Plasma-deposited parylene-like thin films: process and material properties. Surf Coat Technol. 2003;174:124–30. 10.1016/s0257-8972(03)00329-3.

[5] Simon P, Mang S, Hasenhindl A, Gronski W, Greiner A. Poly(p-xylylene) and its derivatives by chemical vapor deposition: synthesis, mechanism, and structure. Macromolecules. 1998;31(25):8775–80. 10.1021/ma9808070.

[6] Rapp BE, Voigt A, Dirschka M, Länge K. Deposition of ultrathin parylene C films in the range of 18 nm to 142 nm: controlling the layer thickness and assessing the closeness of the deposited films. Thin Solid Films. 2012;520(15):4884–8. 10.1016/j.tsf.2012.03.035.

[7] Hu SM, Lee CY, Ramli TC, Christy J, Chang YM, Lee KJ, et al. Vapor-phase synthesis of poly(para-xylylene): from coatings to porous and hierarchical materials. Adv Funct Mater. 2024;34(47):2313511. 10.1002/adfm.202313511.

[8] Golda-Cepa M, Engvall K, Hakkarainen M, Kotarba A. Recent progress on parylene C polymer for biomedical applications: a review. Prog Org Coat. 2020;140:105493. 10.1016/j.porgcoat.2019.105493.

[9] Song Z, Im JH, Ko H, Park JH, Lee GY, Kang MJ, et al. Plasma deposition of parylene-C film. Mater Today Commun. 2021;26:101834. 10.1016/j.mtcomm.2020.101834.

[10] Song ZQ, Jung JY, Kim TH, Park JH, Song HW, Kang MJ, et al. Plasma deposition of parylene-like films with chemical functional groups for immunoassays. ACS Appl Mater Interfaces. 2024;16(46):63255–67. 10.1021/acsami.4c13474. 39501653

[11] Hsu JM, Rieth L, Kammer S, Orthner M, Solzbacher F. Effect of thermal and deposition processes on surface morphology, crystallinity, and adhesion of Parylene-C. Sens Mater. 2008;20(2):87–102.

[12] Ebrahimibasabi S, Golshahi M, Shahraki N, Shabestari DT, Sajjadi M, Hashemi S, et al. Designing parylene coating for implantable brain-machine interfaces. RSC Adv. 2025;15(33):26660–72. 10.1039/d5ra04683a. PMC12301961 40727297

[13] Song JS, Lee S, Jung SH, Cha GC, Mun MS. Improved biocompatibility of parylene-C films prepared by chemical vapor deposition and the subsequent plasma treatment. J Appl Polym Sci. 2009;112(6):3677–85. 10.1002/app.29774.

[14] Kim BJ, Meng E. Micromachining of parylene C for bioMEMS. Polym Adv Technol. 2016;27(5):564–76. 10.1002/pat.3729.

[15] Alger M. Polymer Science Dictionary. 3rd ed. Dordrecht: Springer; 2017. 10.1007/978-94-024-0893-5.

[16] Diener electronic. The properties of parylene. Available from: https://www.plasma.com/en/parylene-technology/. Accessed 12 Mar 2026.

[17] CAS Common Chemistry. CAS, a division of the American Chemical Society, n.d. Dichloro[2.2]paracyclophane. Available from: https://commonchemistry.cas.org/detail?cas_rn=28804-46-8. Accessed 24 Mar 2026.

[18] CAS Common Chemistry. CAS, a division of the American Chemical Society, n.d. Tetrachlorotricyclo[8.2.2.24,7]hexadeca-4,6,10,12,13,15-hexaene. Available from: https://commonchemistry.cas.org/detail?cas_rn=30501-29-2. Accessed 24 Mar 2026.

[19] Specialty Coating Systems. SCS parylene properties. Available from: https://scscoatings.com/parylene-coatings/properties/. Accessed 25 Mar 2026.

[20] Specialty Coating Systems. Our history. Available from: https://scscoatings.com/scs-coating-history/. Accessed 25 Mar 2026.

[21] Kisco. diX coatings. Available from: https://www.kisco-net.com/business/dix-coating/. Accessed 25 Mar 2026.

[22] Daisan Kasai. よくあるご質問 [Frequently Asked Questions] (translated by Google Translate). Available from: https://www.dsk-goi.com/dix/dix_faq/. Accessed 25 Mar 2026.

[23] Ma X, Li T, Yu LL, Liu P, Yang WY, Li W. From hydrophobic to hydrophilic: enhancing surface properties of parylene F coatings through oxygen plasma modification. Colloid Surf B-Biointerfaces. 2026;258:115248. 10.1016/j.colsurfb.2025.115248. 41202454

[24] Zhao H, Venton BJ. Recent applications of parylene and pyrolyzed parylene in sensors and devices: a review. Anal Bioanal Chem. 2026;418(2):499–512. 10.1007/s00216-025-06150-1. PMC12783324 41107471

[25] Tan CP, Craighead HG. Surface engineering and patterning using parylene for biological applications. Materials (Basel). 2010;3(3):1803–32. 10.3390/ma3031803.

[26] Chen HY, Lahann J. Designable biointerfaces using vapor-based reactive polymers. Langmuir. 2011;27(1):34–48. 10.1021/la101623n. 20590103

[27] Wu MG, Hsu HL, Hsiao KW, Hsieh CC, Chen HY. Vapor-deposited parylene photoresist: a multipotent approach toward chemically and topographically defined biointerfaces. Langmuir. 2012;28(40):14313–22. 10.1021/la302099y. 22966949

[28] Hermanson GT. Bioconjugate techniques. 2nd ed. USA: Academic Press; 2008. 10.1016/B978-0-12-370501-3.X0001-X.

[29] Raos BJ, Simpson MC, Doyle CS, Graham ES, Unsworth CP. Evaluation of parylene derivatives for use as biomaterials for human astrocyte cell patterning. PLoS ONE. 2019;14(6):e0218850. 10.1371/journal.pone.0218850. PMC6592558 31237927

[30] Nowlin TE, Fosssmith D, Cieloszyk GS. Thermal oxidative stability of poly-p-xylylenes. J Polym Sci Pol Chem. 1980;18(7):2103–19. 10.1002/pol.1980.170180707.

[31] Monk DJ, Toh HS, Wertz J. Oxidative degradation of parylene C (poly(monochloro-para-xylylene)) thin films on bulk micromachined piezoresistive silicon pressure sensors. Sens Mater. 1997;9(5):307–19.

[32] Bera M, Rivaton A, Gandon C, Gardette JL. Comparison of the photodegradation of parylene C and parylene N. Eur Polym J. 2000;36(9):1765–77. 10.1016/s0014-3057(99)00259-1.

[33] Pruden KG, Sinclair K, Beaudoin S. Characterization of parylene-N and parylene-C photooxidation. J Polym Sci Pol Chem. 2003;41(10):1486–96. 10.1002/pola.10681.

[34] Trantidou T, Prodromakis T, Toumazou C. Oxygen plasma induced hydrophilicity of parylene-C thin films. Appl Surf Sci. 2012;261:43–51. 10.1016/j.apsusc.2012.06.112.

[35] Bae J, Lee IJ. Wettability, interface structure, and chemistry in functionalized poly(chloro-para-xylylene) films. Appl Surf Sci. 2014;303:344–9. 10.1016/j.apsusc.2014.02.184.

[36] Golda-Cepa M, Aminlashgari N, Hakkarainen M, Engvall K, Kotarba A. LDI-MS examination of oxygen plasma modified polymer for designing tailored implant biointerfaces. RSC Adv. 2014;4(50):26240–3. 10.1039/c4ra02656j.

[37] Liaqat U, Ko H, Suh H, Lee M, Pyun JC. UV-irradiated parylene surfaces for proliferation and differentiation of PC-12 cells. Enzyme Microb Technol. 2017;97:1–10. 10.1016/j.enzmictec.2016.10.016. 28010765

[38] Kim TH, Lee JS, Jo HH, Park YS, Yun MJ, Song ZQ, et al. Modified parylene-N films as chemical microenvironments for differentiation and spheroid formation of osteoblast cells. Sci Rep. 2020;10(1):15219. 10.1038/s41598-020-71322-1. PMC7495472 32938961

[39] Hoshino T, Saito I, Kometani R, Samejima K, Matsui S, Suzuki T, et al. Improvement of neuronal cell adhesiveness on parylene with oxygen plasma treatment. J Biosci Bioeng. 2012;113(3):395–8. 10.1016/j.jbiosc.2011.11.003. 22138385

[40] Ko H, Choi YH, Chang SY, Lee GY, Song HW, Chang YW, et al. Surface modification of parylene-N with UV-treatment to enhance the protein immobilization. Eur Polym J. 2015;68:36–46. 10.1016/j.eurpolymj.2015.04.016.

[41] Länge K, Grimm S, Rapp M. Chemical modification of parylene C coatings for SAW biosensors. Sens Actuators B Chem. 2007;125(2):441–6. 10.1016/j.snb.2007.02.039.

[42] Herrera-Alonso M, McCarthy TJ. Chemical surface modification of poly(p-xylylene) thin films. Langmuir. 2004;20(21):9184–9. 10.1021/la049025s. 15461504

[43] Satheeshkumar C, Jung BJ, Jang H, Lee W, Seo M. Surface modification of parylene C film via Buchwald-Hartwig amination for organic solvent-compatible and flexible microfluidic channel bonding. Macromol Rapid Commun. 2021;42(8):2000520. 10.1002/marc.202000520. 33225498

[44] Wahjudi PN, Oh JH, Salman SO, Seabold JA, Rodger DC, Tai YC, et al. Improvement of metal and tissue adhesion on surface-modified parylene C. J Biomed Mater Res A. 2009;89A(1):206–14. 10.1002/jbm.a.31929. 18431781

[45] Zhang CH, Thompson ME, Markland FS, Swenson S. Chemical surface modification of parylene C for enhanced protein immobilization and cell proliferation. Acta Biomater. 2011;7(10):3746–56. 10.1016/j.actbio.2011.06.003. 21689793

[46] Barié N, Rapp M. Covalent bound sensing layers on surface acoustic wave (SAW) biosensors. Biosens Bioelectron. 2001;16(9–12):979–87. 10.1016/s0956-5663(01)00198-1. 11679278

[47] Länge K, Bender F, Voigt A, Gao H, Rapp M. A surface acoustic wave biosensor concept with low flow cell volumes for label-free detection. Anal Chem. 2003;75(20):5561–6. 10.1021/ac0207574. 14710839

[48] Plank M, Berardi A, Welle A, Sauter E, Krolla P, Haret C, et al. Photo-Arbuzov reactions as a broadly applicable surface modification strategy. Adv Funct Mater. 2024;34(47):2403408. 10.1002/adfm.202403408.

[49] O’Grady BJ, Geuy MD, Kim H, Balotin KM, Allchin ER, Florian DC, et al. Rapid prototyping of cell culture microdevices using parylene-coated 3D prints. Lab Chip. 2021;21(24):4814–22. 10.1039/d1lc00744k. PMC8717820 34787148

[50] Golda-Cepa M, Kulig W, Cwiklik L, Kotarba A. Molecular dynamics insights into water-parylene C interface: relevance of oxygen plasma treatment for biocompatibility. ACS Appl Mater Interfaces. 2017;9(19):16685–93. 10.1021/acsami.7b03265. 28459527

[51] Chang TY, Yadav VG, De Leo S, Mohedas A, Rajalingam B, Chen CL, et al. Cell and protein compatibility of parylene-C surfaces. Langmuir. 2007;23(23):11718–25. 10.1021/la7017049. 17915896

[52] Gholizadeh S, Lincoln DM, Allahyari Z, Widom LP, Carter RN, Gaborski TR. Optimization of parylene C and parylene N thin films for use in cellular co-culture and tissue barrier models. Sci Rep. 2023;13(1):4262. 10.1038/s41598-023-31305-4. PMC10015097 36918711

[53] Chou CM, Hsieh KC, Chung CJ, He JL. Preparation of plasma-polymerized para-xylene as an alternative to parylene coating for biomedical surface modification. Surf Coat Technol. 2010;204(9–10):1631–6. 10.1016/j.surfcoat.2009.10.038.

[54] Länge K. Bulk and surface acoustic wave sensor arrays for multi-analyte detection: a review. Sensors. 2019;19(24):5382. 10.3390/s19245382. PMC6960530 31817599

[55] Kirkendall C, Kwon JW. Femtogram mass resolution in a liquid environment using a low loss vacuum-gapped quartz crystal resonator. Lab Chip. 2011;11(4):596–8. 10.1039/c0lc00367k. 21103592

[56] Yang YC, Zhang W, Guo Z, Zhang ZQ, Zhu HN, Yan RH, et al. Stability enhanced, repeatability improved Parylene-C passivated on QCM sensor for aPTT measurement. Biosens Bioelectron. 2017;98:41–6. 10.1016/j.bios.2017.06.021. 28649023

[57] Yao J, Feng B, Zhang ZQ, Li CY, Zhang W, Guo Z, et al. Blood coagulation testing smartphone platform using quartz crystal microbalance dissipation method. Sensors (Basel). 2018;18(9):3073. 10.3390/s18093073. PMC6164724 30217015

[58] Han HC, Chang YR, Hsu WL, Chen CY. Application of parylene-coated quartz crystal microbalance for on-line real-time detection of microbial populations. Biosens Bioelectron. 2009;24(6):1543–9. 10.1016/j.bios.2008.07.033. 18819789

[59] Barié N, Stahl U, Rapp M. Vacuum-deposited wave-guiding layers on STW resonators based on LiTaO3 substrate as love wave sensors for chemical and biochemical sensing in liquids. Ultrasonics. 2010;50(6):606–12. 10.1016/j.ultras.2009.12.006. 20092864

[60] Bender F, Länge K, Voigt A, Rapp M. Improvement of surface acoustic wave gas and biosensor response characteristics using a capacitive coupling technique. Anal Chem. 2004;76(13):3837–40. 10.1021/ac035019+. 15228363

[61] Rapp BE, Voigt A, Dirschka M, Rapp M, Länge K. Surface acoustic wave resonator chip setup for the elimination of interfering conductivity responses. Micromachines (Basel). 2024;15(4):501. 10.3390/mi15040501. PMC11052277 38675312

[62] Gizeli E, Goddard NJ, Lowe CR, Stevenson AC. A Love plate biosensor utilising a polymer layer. Sens Actuator B-Chem. 1992;6(1–3):131–7. 10.1016/0925-4005(92)80044-x.

[63] Gizeli E, Bender F, Rasmusson A, Saha K, Josse F, Cernosek R. Sensitivity of the acoustic waveguide biosensor to protein binding as a function of the waveguide properties. Biosens Bioelectron. 2003;18(11):1399–406. 10.1016/s0956-5663(03)00080-0. 12896842

[64] Bender F, Länge K, Barié N, Kondoh J, Rapp M. On-line monitoring of polymer deposition for tailoring the waveguide characteristics of love-wave biosensors. Langmuir. 2004;20(6):2315–9. 10.1021/la035674c. 15835690

[65] Länge K, Rapp BE, Rapp M. Surface acoustic wave biosensors: a review. Anal Bioanal Chem. 2008;391(5):1509–19. 10.1007/s00216-008-1911-5. 18265962

[66] Kong H, Li CY, Guo Z, Zhang W, Yao J, Zhu HN, et al. Sensitivity improved with parylene-C passivized on Lamb wave sensor for aPTT measurement through monitoring whole blood reaction. Sens Actuator B-Chem. 2019;285:479–86. 10.1016/j.snb.2019.01.053.

[67] Wu H, Zu H, Wang Q-M, Zhao G, Wang JH-C. Label-free detection of protein released during platelet activation by CNT-enhanced love mode SAW sensors. 2014 IEEE International Ultrasonics Symposium; 03–06 September 2014 Chicago, IL, USA IEEE; 2014. 10.1109/ULTSYM.2014.0378 PMC4431625 25984268

[68] Wu HY, Zu HF, Wang JHC, Wang QM. A study of Love wave acoustic biosensors monitoring the adhesion process of tendon stem cells (TSCs). Eur Biophys J Biophys Lett. 2019;48(3):249–60. 10.1007/s00249-019-01349-4. 30783690

[69] Wu H, Zu H, Wang Q-M, Wang JH-C. A Love wave biosensor with a phononic wave guiding layer for VEGF detection in selective platelet activation. 2017 IEEE International Ultrasonics Symposium (IUS); 06–09 September 2017; Washington, DC, USA IEEE; 2017. 10.1109/ULTSYM.2017.8092842

[70] Hohmann S, Kögel S, Brunner Y, Schmieg B, Ewald C, Kirschhöfer F, et al. Surface acoustic wave (SAW) resonators for monitoring conditioning film formation. Sensors. 2015;15(5):11873–88. 10.3390/s150511873. PMC4481949 26007735

[71] Gruhl FJ, Länge K. Surface modification of an acoustic biosensor allowing the detection of low concentrations of cancer markers. Anal Biochem. 2012;420(2):188–90. 10.1016/j.ab.2011.10.006. 22033293

[72] Gruhl FJ, Länge K. Influence of surface preparation parameters on the signal response of an acoustic biosensor for the detection of a breast cancer marker. IEEE Sens J. 2012;12(6):1647–8. 10.1109/jsen.2011.2175722.

[73] Berger M, Welle A, Gottwald E, Rapp M, Länge K. Biosensors coated with sulfated polysaccharides for the detection of hepatocyte growth factor/scatter factor in cell culture medium. Biosens Bioelectron. 2010;26(4):1706–9. 10.1016/j.bios.2010.07.065. 20719493

[74] Pomowski A, Baricham C, Rapp BE, Matern A, Länge K. Acoustic biosensors coated with phosphorylcholine groups for label-free detection of human C-reactive protein in serum. IEEE Sens J. 2015;15(8):4388–92. 10.1109/jsen.2015.2419278.

[75] Gruhl FJ, Länge K. Surface acoustic wave (SAW) biosensor for rapid and label-free detection of penicillin G in milk. Food Anal Meth. 2014;7(2):430–7. 10.1007/s12161-013-9642-4.

[76] Erofeev A, Antifeev I, Bolshakova A, Bezprozvanny I, Vlasova O. In vivo penetrating microelectrodes for brain electrophysiology. Sensors. 2022;22(23):9085. 10.3390/s22239085. PMC9735502 36501805

[77] Rafi H, Zestos AG. Multiplexing neurochemical detection with carbon fiber multielectrode arrays using fast-scan cyclic voltammetry. Anal Bioanal Chem. 2021;413(27):6715–26. 10.1007/s00216-021-03526-x. PMC8551007 34259877

[78] Mariello M, Kim K, Wu KL, Lacour SP, Leterrier Y. Recent advances in encapsulation of flexible bioelectronic implants: materials, technologies, and characterization methods. Adv Mater. 2022;34(34):2201129. 10.1002/adma.202201129. 35353928

[79] Eyimegwu F, Zhao H, Shrestha K, Surendran D, Lavrik NV, Venton BJ. Pyrolyzed parylene electrodes for detection of tryptophan, tyrosine, and gonadotropin-releasing hormone. ACS Meas Sci Au. 2026;6(1):214–23. 10.1021/acsmeasuresciau.5c00165. PMC12921596 41727377

[80] Zheng QL, Xu Y. Design and optimization of parylene nanomechanical cantilevers with integrated piezoresistors for surface-stress based biochemical sensing. Microsyst Technol. 2011;17(3):351–9. 10.1007/s00542-011-1275-1.

[81] Maheshwari N, Chatterjee G, Vinchurkar M, Gupta G, Kshirsagar H, Narsimhan KL, Rao VR. Parylene-C encapsulation for polymeric cantilever stability. 2015 Symposium on Design, Test, Integration and Packaging of MEMS/MOEMS (DTIP); 27–30 April 2015; Montpellier, France: IEEE; 2015. 10.1109/DTIP.2015.7161032

[82] Satyanarayana S, McCormick DT, Majumdar A. Parylene micro membrane capacitive sensor array for chemical and biological sensing. Sens Actuators B Chem. 2006;115(1):494–502. 10.1016/j.snb.2005.10.013.

[83] Choi YJ, Takahashi T, Taki M, Sawada K, Takahashi K. Label-free attomolar protein detection using a MEMS optical interferometric surface-stress immunosensor with a freestanding PMMA/parylene-C nanosheet. Biosens Bioelectron. 2021;172:112778. 10.1016/j.bios.2020.112778. 33157412

[84] Choi YH, Lee GY, Ko H, Chang YW, Kang MJ, Pyun JC. Development of SPR biosensor for the detection of human hepatitis B virus using plasma-treated parylene-N film. Biosens Bioelectron. 2014;56:286–94. 10.1016/j.bios.2014.01.035. 24518301

[85] Choi YH, Ko H, Lee GY, Chang SY, Chang YW, Kang MJ, et al. Development of a sensitive SPR biosensor for C-reactive protein (CRP) using plasma-treated parylene-N film. Sens Actuator B-Chem. 2015;207:133–8. 10.1016/j.snb.2014.10.012.

[86] Lv XQ, Ma ZT, Jiang WH, Huang CC, Deng J, Zhang H, et al. Ultrathin parylene C-based sensitivity-gain nanoplasmonic sensor integrated on VCSEL for Aβ 42 detection. Biosens Bioelectron. 2024;254:116220. 10.1016/j.bios.2024.116220. 38518564

[87] Miksa BJ, Sochacki M, Sroka-Bartnicka A, Uznanski P, Nosal A, Potrzebowski MJ. Application of parylene for surface (polymer) enhanced laser desorption/ionization of synthetic polymers. Rapid Commun Mass Spectrom. 2013;27(7):767–72. 10.1002/rcm.6516. 23495023

[88] Noh JY, Kim MJ, Park JM, Yun TG, Kang MJ, Pyun JC. Quantitative analysis of vitamin D using m/MALDI-TOF mass spectrometry based on a parylene matrix chip. J Anal Sci Technol. 2022;13(1):3. 10.1186/s40543-021-00313-2.

[89] Kim JI, Park JM, Kang MJ, Pyun JC. Parylene-matrix chip for small molecule analysis using matrix-assisted laser desorption/ionization time-of-flight mass spectrometry. Rapid Commun Mass Spectrom. 2014;28(3):274–80. 10.1002/rcm.6782. 24375878

[90] Park JM, Kim JI, Song HW, Noh JY, Kang MJ, Pyun JC. Highly sensitive bacterial susceptibility test against penicillin using parylene-matrix chip. Biosens Bioelectron. 2015;71:306–12. 10.1016/j.bios.2015.04.059. 25919811

[91] Park JM, Kim JI, Noh JY, Kim M, Kang MJ, Pyun JC. A highly sensitive carbapenemase assay using laser desorption/ionization mass spectrometry based on a parylene-matrix chip. Enzyme Microb Technol. 2017;104:56–68. 10.1016/j.enzmictec.2017.05.010. 28648181

[92] Kim JI, Noh JY, Kim M, Park JM, Song HW, Kang MJ, et al. Newborn screening by matrix-assisted laser desorption/ionization mass spectrometry based on parylene-matrix chip. Anal Biochem. 2017;530:31–9. 10.1016/j.ab.2017.04.021. 28465033

[93] Park JM, Noh JY, Kim MJ, Yun TG, Lee SG, Chung KS, et al. MALDI-TOF mass spectrometry based on parylene-matrix chip for the analysis of lysophosphatidylcholine in sepsis patient sera. Anal Chem. 2019;91(22):14719–27. 10.1021/acs.analchem.9b04019. 31621295

[94] Park JM, Kim MJ, Noh JY, Yun TG, Kang MJ, Lee SG, et al. Simultaneous analysis of multiple cancer biomarkers using MALDI-TOF mass spectrometry based on a parylene-matrix chip. J Am Soc Mass Spectrom. 2020;31(4):917–26. 10.1021/jasms.9b00102. 32154716

[95] Noh JY, Kim MJ, Yun TG, Kang MJ, Pyun JC. Simultaneous analysis of acylcarnitines using MALDI-TOF mass spectrometry based on a parylene matrix chip. Analyst. 2022;147(22):5044–53. 10.1039/d2an01199a. 36196954

[96] Buchwalder S, Borzì A, Leon JD, Bourgeois F, Nicolier C, Nicolay S, et al. Thermal analysis of parylene thin films for barrier layer applications. Polymers. 2022;14(17):3677. 10.3390/polym14173677. PMC9527014 36080750

[97] Plasma Parylene Systems. Technical Data Sheet Polyparylene 2023. Available from: https://plasmaparylene.de/wp-content/uploads/2023/10/Technical-Data-Sheet_Polyparylene_20230111.pdf. Accessed 12 Mar 2026.

[98] Lu B, Zheng SY, Quach BQ, Tai YC. A study of the autofluorescence of parylene materials for μTAS applications. Lab Chip. 2010;10(14):1826–34. 10.1039/b924855b. 20431822

[99] Diener electronic. The properties of parylene: characteristics of the parylene types. Available from: https://www.plasma.com/en/parylene-technology/. Accessed 12 Mar 2026.

[100] United States Environmental Protection Agency. PFAS structures in DSSTox (update August 2022) 2022. Available from: https://comptox.epa.gov/dashboard/chemical-lists/PFASSTRUCTV5. Accessed 12 Mar 2026.

[101] United States Environmental Protection Agency. Tricyclo(8.2.2.24,7)hexadeca-4,6,10,12,13,15-hexaene, 2,2,3,3,8,8,9,9,-octafluoro- | 3345–29–7 | DTXSID30187095 2025. Available from: https://comptox.epa.gov/dashboard/chemical/details/DTXSID30187095. Accessed 12 Mar 2026.

[102] United States Environmental Protection Agency. Parylene F dimer | 1785–64–4 | DTXSID501338087 2025. Available from: https://comptox.epa.gov/dashboard/chemical/details/DTXSID501338087. Accessed 12 Mar 2026.

[103] Bilela LL, Matijosyte I, Krutkevicius J, Alexandrino DAM, Safarik I, Burlakovs J, et al. Impact of per- and polyfluorinated alkyl substances (PFAS) on the marine environment: raising awareness, challenges, legislation, and mitigation approaches under the One Health concept. Mar Pollut Bull. 2023;194:115309. 10.1016/j.marpolbul.2023.115309. 37591052

[104] Raos BJ, Graham ES, Murray AF, Simpson MC, Unsworth CP. Investigating parylene-HT as a substrate for human cell patterning. 38th Annual International Conference of the IEEE Engineering in Medicine and Biology Society (EMBC); 16–20 August 2016 Orlando, FL, USA: IEEE; 2016. p. 141–4. 10.1109/EMBC.2016.7590660 28268299

[105] Akhtar M, van den Driesche S, Bödecker A, Vellekoop MJ. Long-term storage of droplets on a chip by parylene AF4 coating of channels. Sens Actuator B-Chem. 2018;255:3576–84. 10.1016/j.snb.2017.08.032.

[106] Han B, Wang H, Bing W, Jin HC. Bacterial adhesion properties of parylene C and D deposited on polydimethylsiloxane. New J Chem. 2022;46(18):8773–8. 10.1039/d1nj06223a.

[107] Bing W, Cai YJ, Jin HC, Tian L, Tian LM, Yin Y, et al. An antiadhesion and antibacterial platform based on parylene F coatings. Prog Org Coat. 2021;151:106021. 10.1016/j.porgcoat.2020.106021.

[108] Tian LM, Yin Y, Zhao J, Jin HC, Shang YG, Yan SX, et al. Parylene F coatings for combating marine biofouling. Mater Lett. 2021;285:129141. 10.1016/j.matlet.2020.129141.

[109] Xu H, Yang Z, Guo YC, Xu QM, Dou ST, Zhang P, et al. Copolymerization of parylene C and parylene F to enhance adhesion and thermal stability without coating performance degradation. Polymers. 2023;15(5):1249. 10.3390/polym15051249. PMC10007139 36904490

[110] Ahmed T, van den Driesche S, Bafna JA, Oellers M, Hemmler R, Gall K, et al. Rapid lipid bilayer membrane formation on parylene coated apertures to perform ion channel analyses. Biomed Microdevices. 2020;22(2):32. 10.1007/s10544-020-0473-y. PMC7192868 32355998

[111] Ahmed T, Bafna JA, Hemmler R, Gall K, Wagner R, Winterhalter M, et al. Silicon nitride-based micro-apertures coated with parylene for the investigation of pore proteins fused in free-standing lipid bilayers. Membranes. 2022;12(3):309. 10.3390/membranes12030309. PMC8954132 35323784

[112] Rodger DC, Fong AJ, Wen L, Ameri H, Ahuja AK, Gutierrez C, et al. Flexible parylene-based multielectrode array technology for high-density neural stimulation and recording. Sens Actuator B-Chem. 2008;132(2):449–60. 10.1016/j.snb.2007.10.069.

[113] Zátonyi A, Madarász M, Szabó A, Lorincz T, Hodován R, Rózsa B, et al. Transparent, low-autofluorescence microECoG device for simultaneous Ca2+ imaging and cortical electrophysiology in vivo. J Neural Eng. 2020;17(1):016062. 10.1088/1741-2552/ab603f. 31822640

[114] Madarász M, Fedor FZ, Fekete Z, Rózsa B. Immunohistological responses in mice implanted with parylene HT - ITO ECoG devices. Front Neurosci. 2023;17:1209913. 10.3389/fnins.2023.1209913. PMC10513038 37746144

[115] Buchwalder S, Hersberger M, Rebl H, Seemann S, Kram W, Hogg A, et al. An evaluation of parylene thin films to prevent encrustation for a urinary bladder pressure MEMS sensor system. Polymers. 2023;15(17):3559. 10.3390/polym15173559. PMC10490164 37688185

[116] Afshari E, Houri S, Verplancke R, Rochus V, Cauwe M, Gijsenbergh P, et al. Biocompatible and hermetic encapsulation of PMUTs: effects of parylene F-VT4 and ALD stacks on membrane vibration and acoustic performance. Sensors. 2025;25(13):4074. 10.3390/s25134074. PMC12251638 40648330

[117] Kato YX, Saito I, Takano H, Mabuchi K, Hoshino T. Comparison of neuronal cell adhesiveness of materials in the diX (Parylene) family. Neurosci Lett. 2009;464(1):26–8. 10.1016/j.neulet.2009.08.007. 19664682

[118] Hoshino T, Saito I, Takano H, Samejima K, Mabuchi K, Kato YX. Neurite outgrowth of PC12 cells on diX (parylene) family materials. Biotechnol Prog. 2012;28(2):587–90. 10.1002/btpr.739. 22052685

[119] St-Georges-Robillard A, Ruiz JC, Petit A, Wang HT, Mwale F, Elkin B, et al. Adhesion of U-937 monocytes on different amine-functionalised polymer surfaces. Plasma Process Polym. 2012;9(3):243–52. 10.1002/ppap.201100128.

[120] Wertheimer MR, St-Georges-Robillard A, Lerouge S, Mwale F, Elkin B, Oehr C, et al. Amine-rich organic thin films for cell culture: possible electrostatic effects in cell-surface interactions. Jpn J Appl Phys. 2012;51(11):11PJ04. 10.1143/jjap.51.11pj04.

[121] Liaqat U, Ko H, Suh H, Lee MS, Pyun JC. Surface modification of parylene-N films for the culture of osteoblast-like cells (MG-63). Appl Surf Sci. 2016;378:277–85. 10.1016/j.apsusc.2016.03.218.

[122] Tozawa H, Maekawa T, Kimura H, Fujii T. A novel effect of parylene-based surface coating on HepG2 cell function. Mater Sci Eng C-Mater Biol Appl. 2015;46:190–4. 10.1016/j.msec.2014.10.030. 25491976

[123] Kim T-H, Song Z, Jung J, Sung J-S, Kang M-J, Shim W-B, et al. Functionalized parylene films for enhancement of antibody production by hybridoma cells. ACS Appl Bio Mater. 2023;6(9):3726–38. 10.1021/acsabm.3c00417. 37647153

[124] Miwa J, Suzuki Y, Kasagi N. Adhesion-based cell sorter with antibody-coated amino-functionalized-parylene surface. J Microelectromech Syst. 2008;17(3):611–22. 10.1109/jmems.2008.921706.

[125] Suzuki Y, Miwa J-I. High-density antibody-immobilized surface for adhesion-based cell separation. Proc 10th IEEE Int Conf Nanotechnol 2010:80–3. 10.1109/NANO.2010.5698081

[126] Ko H, Lee EH, Lee GY, Kim J, Jeon BJ, Kim MH, et al. One step immobilization of peptides and proteins by using modified parylene with formyl groups. Biosens Bioelectron. 2011;30(1):56–60. 10.1016/j.bios.2011.08.026. 21945140

[127] Ko H, Lee GY, Jeon BJ, Pyun JC. Fluorescence immunoassay of anti-cyclic citrulinated peptide (CCP) autoantibodies by using parylene-H film. BioChip J. 2011;5(3):242–5. 10.1007/s13206-011-5308-8.

[128] Yoo G, Park M, Lee EH, Jose J, Pyun JC. Immobilization of E-coli with autodisplayed Z-domains to a surface-modified microplate for immunoassay. Anal Chim Acta. 2011;707(1):142–7. 10.1016/j.aca.2011.09.004. 22027131

[129] Tashiro M, Tejero R, Zimmerman DE, Celda B, Nilsson B, Montelione GT. High-resolution solution NMR structure of the Z domain of staphylococcal protein A. J Mol Biol. 1997;272(4):573–90. 10.1006/jmbi.1997.1265. 9325113

[130] Han H, Pyun JC, Yoo H, Seo HS, Jung BH, Yoo YS, et al. Highly sensitive immunoassay for the diagnosis of acute myocardial infarction using silica spheres encapsulating a quantum dot layer. Anal Chem. 2014;86(20):10157–63. 10.1021/ac502412x. 25257659

[131] Jeon BJ, Kim MH, Pyun JC. Application of a functionalized parylene film as a linker layer of SPR biosensor. Sens Actuator B-Chem. 2011;154(2):89–95. 10.1016/j.snb.2010.01.035.

[132] Shin EJ, Lee WG, Moon SJ. Characterization of a novel antibody immobilization combining protein G with parylene-H for surface plasmon resonance immunosensors. Microsyst Technol. 2016;22(8):2093–9. 10.1007/s00542-015-2534-3.

[133] Lee EH, Yoo G, Jose J, Kang MJ, Song SM, Pyun JC. SPR biosensor based on immobilized *E.coli* cells with autodisplayed Z-domains. Biochip J. 2012;6(3):221–8. 10.1007/s13206-012-6304-3.

[134] Kim HR, Bong JH, Jung J, Sung JS, Kang MJ, Park JG, et al. An on-chip chemiluminescent immunoassay for bacterial detection using in situ-synthesized cadmium sulfide nanowires with passivation layers. Biochip J. 2020;14(3):268–78. 10.1007/s13206-020-4305-1.

[135] Jung HW, Chang YW, Lee GY, Cho S, Kang MJ, Pyun JC. A capacitive biosensor based on an interdigitated electrode with nanoislands. Anal Chim Acta. 2014;844:27–34. 10.1016/j.aca.2014.07.006. 25172812

[136] Tsai CN, Lee CY, Chen HY, Hsieh BC. Parylene double-layer coated screen-printed carbon electrode for label-free and reagentless capacitive aptasensing of gliadin. ACS Sens. 2024;9(7):3689–96. 10.1021/acssensors.4c00875. PMC11287748 38982801

[137] Park M, Kim J, Kim K, Pyun JC, Sung GY. Parylene-coated polytetrafluoroethylene-membrane-based portable urea sensor for real-time monitoring of urea in peritoneal dialysate. Sensors. 2019;19(20):4560. 10.3390/s19204560. PMC6832945 31635189

[138] Kim J-I, Lee G-Y, Ko H, Kang M-J, Pyun J-C. Covalent protein immobilization with a parylene-H film for matrix-assisted laser desorption/ionization time-of-flight mass spectrometry. Rapid Commun Mass Spectrom. 2013;27(10):1149–54. 10.1002/rcm.6555. 23592120

[139] Luu T, Gristwood K, Knight JC, Jörg M. Click chemistry: reaction rates and their suitability for biomedical applications. Bioconjugate Chem. 2024;35(6):715–31. 10.1021/acs.bioconjchem.4c00084. PMC11191409 38775705

[140] Nandivada H, Chen HY, Bondarenko L, Lahann J. Reactive polymer coatings that “click.” Angew Chem-Int Edit. 2006;45(20):3360–3. 10.1002/anie.200600357. 16622893

[141] Tsai MY, Lin CY, Huang CH, Gu JA, Huang ST, Yu JS, et al. Vapor-based synthesis of maleimide-functionalized coating for biointerface engineering. Chem Commun. 2012;48(89):10969–71. 10.1039/c2cc35892a. 23034599

[142] Senkevich JJ, Woods BW, McMahon JJ, Wang PI. Thermomechanical properties of parylene X, a room-temperature chemical vapor depositable crosslinkable polymer. Chem Vapor Depos. 2007;13(1):55–9. 10.1002/cvde.200606541.

[143] Chen HY, Hirtz M, Deng XP, Laue T, Fuchs H, Lahann J. Substrate-independent dip-pen nanolithography based on reactive coatings. J Am Chem Soc. 2010;132(51):18023–5. 10.1021/ja108679m. 21138264

[144] Sun TP, Tai CH, Wu JT, Wu CY, Liang WC, Chen HY. Multifaceted and route-controlled “click” reactions based on vapor-deposited coatings. Biomater Sci. 2016;4(2):265–71. 10.1039/c5bm00417a. 26650976

[145] Tsai YT, Wu CY, Guan ZY, Sun HY, Cheng NC, Yeh SY, et al. Topologically controlled cell differentiation based on vapor-deposited polymer coatings. Langmuir. 2017;33(36):8943–9. 10.1021/acs.langmuir.7b01984. 28825482

[146] Guan ZY, Wu CY, Chen TY, Huang ST, Chiang YC, Chen HY. Clickable and photo-erasable surface functionalities by using vapor-deposited polymer coatings. ACS Biomater Sci Eng. 2019;5(4):1753–61. 10.1021/acsbiomaterials.9b00142. 33405551

[147] Bally F, Cheng K, Nandivada H, Deng XP, Ross AM, Panades A, et al. Co-immobilization of biomolecules on ultrathin reactive chemical vapor deposition coatings using multiple click chemistry strategies. ACS Appl Mater Interfaces. 2013;5(19):9262–8. 10.1021/am401875x. 23888837

[148] Deng XP, Lahann J. A generic strategy for co-presentation of heparin-binding growth factors based on CVD polymerization. Macromol Rapid Commun. 2012;33(17):1459–65. 10.1002/marc.201200343. 22887691

[149] Deng XP, Eyster TW, Elkasabi Y, Lahann J. Bio-orthogonal polymer coatings for co-presentation of biomolecules. Macromol Rapid Commun. 2012;33(8):640–5. 10.1002/marc.201100819. 22351186

[150] Winkler AL, Koenig M, Welle A, Trouillet V, Kratzer D, Hussal C, et al. Bioinstructive coatings for hematopoietic stem cell expansion based on chemical vapor deposition copolymerization. Biomacromol. 2017;18(10):3089–98. 10.1021/acs.biomac.7b00743. 28767236

